# Comprehensive Analysis of the Clinical and Biological Significances of Endoplasmic Reticulum Stress in Diffuse Gliomas

**DOI:** 10.3389/fcell.2021.619396

**Published:** 2021-07-09

**Authors:** Ruoyu Huang, Guanzhang Li, Kuanyu Wang, Zhiliang Wang, Fan Zeng, Huimin Hu, Tao Jiang

**Affiliations:** ^1^Department of Molecular Neuropathology, Beijing Neurosurgical Institute, Capital Medical University, Beijing, China; ^2^Department of Neurosurgery, Beijing Tiantan Hospital, Capital Medical University, Beijing, China; ^3^Chinese Glioma Cooperative Group (CGCG), Beijing, China; ^4^Department of Gamma Knife Center, Beijing Neurosurgical Institute, Capital Medical University, Beijing, China

**Keywords:** glioma, ER stress, signature, prognosis, tumor immune environment

## Abstract

**Background:**

As a critical organelle for protein and lipid synthesis, the dysfunction of endoplasmic reticulum has a significant impact on multiple biological processes of cells. Thus, in this study, we constructed an ER stress-related risk signature to investigate the functional roles of ER stress in gliomas.

**Methods:**

A total of 626 samples from TCGA RNA-seq dataset (training cohort) and 310 samples from CGGA RNA-seq dataset (validation cohort) were enrolled in this study. Clinical information and genomic profiles were also obtained. The ER stress signature was developed by the LASSO regression model. The prognostic value of the risk signature was evaluated by Cox regression, Kaplan-Meier and ROC Curve analyses. Bioinformatics analysis and experiment *in vitro* were performed to explore the biological implication of this signature.

**Results:**

We found that the ER stress-related signature was tightly associated with major clinicopathological features and genomic alterations of gliomas. Kaplan-Meier curve and Cox regression analysis indicated that ER stress activation was an independent prognostic factor for patients with glioma. Besides, we also constructed an individualized prognosis prediction model through Nomogram and ROC Curve analysis. Bioinformatics analysis suggested that ER stress activation also promoted the malignant progression of glioma and participated in the regulation of tumor immune microenvironment, especially the infiltration of macrophages in M2 phase. These results were further validated in IHC analysis and cell biology experiments.

**Conclusion:**

The ER stress activation had a high prognostic value and could serve as a promising target for developing individualized treatment of glioma.

## Introduction

Glioma is the most common and aggressive tumor in central nervous system (Wen and Kesari, 2008; Ricard et al., 2012; Jiang et al., 2016). Despite significant advances in conventional therapies including surgical resection with concomitant radiotherapy and chemotherapy, the clinical prognosis of patients with malignant glioma were still unsatisfied, with a five-year survival rate of 5–13% (Phillips et al., 2006; Nuno et al., 2013; Reifenberger et al., 2017). In past decades, researches were devoted to the targeted drugs and immunotherapy of gliomas and produced very limited success (Osuka and Van Meir, 2017; Cloughesy et al., 2019; Zhao et al., 2019). In order to develop more effective treatment strategies to improve the clinical prognosis of patients, a deeper understanding of the molecular mechanisms underlying the genesis and malignant progression of glioma is imperative. In this study, we focused on the functional roles and prognosis value of endoplasmic reticulum (ER) stress activation in gliomas.

Endoplasmic reticulum stress is imbalance of the ER homeostasis induced by accumulation of unfolded or misfolded proteins and alteration of the Ca^+^ concentration (Adamopoulos et al., 2014, 2016; Leprivier et al., 2015). As a crucial organelle, the dysfunction of ER has significant impact on multiple cellular processes (Gutierrez and Simmen, 2014). Relevant researches proved that ER stress participates in the genesis and malignant progression of multiple human cancers (Joo et al., 2007; Obacz et al., 2017). Besides, as a self-protective mechanism against the exogenous or endogenous stress, ER stress was involved in the proliferation of cancer cells in hypoxia environment and the resistance to radiotherapy or chemotherapy (Yao et al., 2020). However, excessive and sustained ER stress could also trigger programmed cell death or apoptosis. According to former research achievements, ER stress was tightly associated with drug-induced apoptosis in lung carcinoma and glioma cells (Joo et al., 2007; Chang et al., 2020). Moreover, cancer cells could acquire drug resistance and survive chemotherapies through ER stress mediated dormancy and immunosuppression (Hamanaka et al., 2005; Lee et al., 2014; Cubillos-Ruiz et al., 2017). These researches indicated that ER stress might be a valuable target for the treatment of malignant tumors. However, the association of ER stress with the biological characteristics and clinical prognosis of glioma remains unclear, which was worth to investigate thoroughly.

In this study, we divided patients into two subgroups based on the profile of ER stress related genes in The Cancer Genome Atlas (TCGA) and Chinese Glioma Genome Atlas (CGGA) datasets and found that there were significant differences in clinicopathological characteristics and prognosis between stratified patients. Then, we constructed a risk signature to evaluate the ER stress activation status and predict the clinical outcome of glioma patients in TCGA dataset. Kaplan-Meier (K-M) survival curve and Cox regression analysis suggested that the ER stress related signature was a valuable prognostic predictor. Besides, by applying nomogram plots, we developed an individualized prediction model which could accurately predict the 1-, 3- and 5-years survival for glioma patients. Bioinformatics analyses were performed to elucidate the functional roles of ER stress in glioma. Furthermore, the downregulation of ER stress could significantly inhibit the proliferation and migration of glioma cell lines *in vitro*. The IHC analysis also showed that the activation of ER stress was tightly associated with the infiltration of macrophages in M2 phase. The results indicated that the risk signature was tightly associated with malignant biological process and the regulation of tumor immune microenvironment in gliomas.

## Materials and Methods

### Samples and Datasets

A total of 626 glioma samples (including 460 low-grade glioma and 166 GBM samples) with RNA sequencing data and clinical information from the Cancer Genome Atlas (TCGA) dataset^1^ were used as discovery cohort. Similarly, 310 glioma samples (including 172 low-grade glioma and 138 GBM samples) with RNA sequencing data and clinical information were downloaded from CGGA dataset^2^ as validation cohort (Zhao et al., 2017). The establishment and management of CGGA dataset was described in the previous study. The clinical characteristics of patients from TCGA and CGGA datasets were summarized in Table 1 and Supplementary Table 1, respectively. Besides, 270 GBM samples with RNA sequencing data and specific tumor anatomic structure information from Ivy Glioblastoma Atlas Project^3^ were also enrolled in this study (Puchalski et al., 2018). All the RNA sequencing data were log2-transformed for further analysis. This study was approved by Institutional Review Board (IRB) of Beijing Tiantan Hospital Affiliated to Capital Medical University.

**TABLE 1:** Characteristics of patients in cluster 1 and cluster 2 in TCGA dataset.

**Characteristic**	***N***	**Cluster 1**	**Cluster 2**	***p* value**
**Total cases**	626	324	302	
**Gender**				0.0933
Male	362	177	185	
Female	264	147	117	
**Age (years)**				< 0.0001
≤40	241	169	72	
>40	385	155	230	
**Grade**				< 0.0001
II	219	184	35	
III	241	139	102	
IV	166	1	165	
**Subtype**				< 0.0001
Classical	87	2	85	
Mesenchymal	99	2	97	
Proneural	224	165	59	
Neural	104	93	11	
**IDH status**				< 0.0001
Mutation	383	305	78	
Wildtype	234	18	216	
**MGMT promoter**				< 0.0001
Methylation	436	291	145	
Unmethylation	153	32	121	
**1p19q**				< 0.0001
Codel	152	144	8	
Intact	468	180	288	

### Consensus Clustering and Construction of Signature

Two ER stress-related gene sets (GO RESPONSE TO ENDOPLASMIC RETICULUM STRESS and GO REGULATION OF RESPONSE TO ENDOPLASMIC RETICULUM STRESS) were downloaded from Molecular Signature Database v7.0 (MSigDB,^4^). Overlapped genes were removed and the acquired ER stress-related gene set contained 272 genes. Among them, 256 genes could be found in TCGA dataset (Supplementary Table 2). Then, we performed univariate Cox analysis with these genes in all glioma patients from TCGA dataset and found that there were 208 genes within the list have prognostic value in patients with gliomas (Supplementary Table 3). The least absolute shrinkage and selection operator (LASSO) analysis was employed to identify the most valuable predictive genes (Bovelstad et al., 2007; Hu et al., 2017). The genes and their coefficients were determined by the best penalty parameter λ associated with the smallest 10-fold cross validation (Tibshirani, 1997; Bovelstad et al., 2007). Then, these obtained genes were employed to construct an optimal risk signature that was determined by a linear combination of their expression levels weighted with regression coefficients from LASSO analysis. The risk score for each sample was calculated as following formula: $R\,i\,s\,k\,s\,c\,o\,r\,e=\sum_{i=1}^{n}\beta \,i\,x\,i$. In this formula, *xi* is the relative expression value of each selected gene and β*i* is the coefficient obtained from LASSO analysis. For validation, the same genes, regression coefficients and formula were applied in CGGA dataset to calculate the risk score.

### Immunohistochemistry

In this study, we performed immunohistochemical (IHC) staining of the protein encoded by signature genes *PDIA4* (Protein disulfide-isomerase A4) and *P4HB* (prolyl 4-hydroxylase, β polypeptide), which were considered as the important biomarker of ER stress. In brief, the sections of glioma tissues were deparaffinized and boiled with ethylenediaminetetraacetic acid (EDTA) antigen retrieval buffer. Then, the sections were incubated with specific primary antibodies overnight at 4°C (anti-*PDIA4*, 1:500 dilutions; anti-*P4HB*, 1:1,000; anti-*CD163*, 1:500; anti-*IBA1*, 1:2,000; Abcam, Cambridge, United Kingdom). All the sections were then incubated with secondary antibodies (ZSGB-Bio, China) at room temperature for 1 h. Finally, the expression levels of each protein in glioma tissues were defined as the portion of positively stained cells against total counted cells in each scan field. IHC staining was quantified by analysis of images from at least five high-power fields. The measure was repeated three times for each section and performed by two experienced pathologists, respectively. Besides, to further validate the results of bioinformatics analysis, IHC staining was also performed to analysis the expression level of the biomarkers of macrophages, *CD163* and *IBA1* (van den Heuvel et al., 1999; Ohsawa et al., 2000). Patients were divided into “low-risk” and “high-risk” groups according to the median risk score in CGGA dataset. The positive expression rates of IHC were compared among glioma patients in the low- and high-risk score groups with a non-parametric test.

### Cell Culture and Transfection

Human glioma cell line U-87MG (U87, RRID:CVCL_0022) and LN229 (RRID:CVCL_0393) were purchased from the Institute of Biochemistry and Cell Biology, Chinese Academy of Sciences. Both of the cell lines were cultured in DMEM medium (Gibco; Thermo Fisher Scientific, United States) supplemented with 10% fetal bovine serum (FBS, Gibco; Thermo Fisher Scientific) at 37°C in a humidified atmosphere of 5% CO_2_. The *P4HB* and *PDIA4* small interference RNA (siRNA) and negative control (NC) were synthesized by RiboBio Co., Ltd. (Guangzhou, China). When the cell density reached 30–50%, LN229 or U87 cell lines was transfected with siRNA or NC (50 nM) at 37°C using the transfection reagent (Polyplus-transfection Co., Ltd., France). After 48 h, fresh medium without siRNA was added to the cells. The sequences for *PDIA4* and *P4HB* siRNA are as follows: *PDIA4* siRNA1 5′-GAGCAAGTTTATAGAAGAA-3′; *PDIA4* siRNA2 5′-GGAAGGCCTTATGACTACA-3′; *PDIA4* siRNA3 5′-GAGTCTTGGTCCTAAATGA-3′. P4HB siRNA1 5′-GTCTGACTATGACGGCAAA-3′; *P4HB* siRNA2 5′-GGCCATCGATGACATACCA-3′; *P4HB* siRNA3 5′-CCATCAAGTTCTTCAGGAA-3′. In this study, all experiments were performed with mycoplasma-free cells and all glioma cell lines have been authenticated using STR profiling.

### Quantitative PCR and Western Blot

Quantitative PCR (qPCR) was performed using the SYBR SuperMix kit (Bio-Rad Laboratories, Inc.) in the 7,500 Fast Real-Time PCR system (Applied Biosystems, United States) according to the manufacturer’s protocols. The relative mRNA expression levels of *PDIA4* and *P4HB* were normalized to *GAPDH* and were calculated using the 2^–ΔΔCq^ method. The primer sequences of *PDIA4*, *P4HB* and *GAPDH* were synthesized by GENEWIZ Co. (Beijing, China). The primer sequences were as follows: *PDIA4*, forward 5′-GGCAGGCTGTAGACTACGAG-3′, and reverse 5′-TTGGTCAACACAAGCGTGAC-3′; *P4HB*, forward 5′-GGTGCTGCGGAAAAGCAAC-3′, and reverse 5′-ACCTGATCTCGGAACCTTCTG-3′; and *GAPDH*, forward 5′-GGAGCGAGATCCCTCCAAAAT-3′, and reverse 5′-GGCTGTTGTCATACTTCTCATGG-3′. Western blot (WB) analysis was performed with rabbit anti-*PDIA4* and anti-*P4HB* polyclonal antibody (1:1,000 and 1:2,000, respectively, Abcam). Mouse anti-*GAPDH* antibody (1:5,000, Abcam) was used for loading control and goat anti-rabbit IgG-HRP (1:5,000, ZSGB-Bio, China) was used as secondary antibodies.

### Cell Migration Assays

After transfection with *PDIA4* siRNA, *P4HB* siRNA or NC siRNA, 1 × 10^5^ cells were seeded into upper chambers of a 24-well transwell chambers (Corning, United States) with 150 μl serum-free medium, while 600 μl medium contained 10% FBS was added into the lower chamber. After that, the mentioned U87 and LN229 cells were incubated at 37°C for 2 or 4 h, respectively. Then, cells on the upper surface of filters were removed with cotton swabs and cells at the bottom surface were fixed and stained with 0.5% crystal violet. Finally, the stained cells were observed using the ×1 and ×10 objectives and measured with × 20 objective.

### Cell Scratch Assays

LN229 and U87 cells were seeded in 6-well plates (1 × 10^5^ cells per well) and incubated at 37°C. After adherence, cells were transfected with specific siRNA for 48 h. Then, the cell monolayer was scraped with a sterile 200-μL pipette tip. After that, the medium in each well were replaced with fresh medium without serum and the “zero point” of migration in each well was photographed under computer-assisted microscopy (ZEISS, Germany). At the end of 48 h incubation, the same scratch area of each well was photographed again.

### Cell Proliferation Assay

Cell proliferation *in vitro* was studied by cell counting kit-8 (CCK-8, Dojindo Laboratories, Japan). After the transfection with specific siRNA, LN229 and U87 cells were plated in 96-well plates and cultured at 37°C for 1–5 days (1 × 10^3^ cells per well). At the same time of each day, 100 μl fresh culture medium and 10 μl CCK-8 reagent were thoroughly mixed and added to each well. After 2 h incubation at 37°C, the absorbances at 450 nm of the medium were measured for further analysis. The measures were performed in triplicate.

### Clonogenic Assays

After transfection with specific siRNA, LN229 and U87 cells were digested and seeded into a 6-well culture plate (400 cells per well). Plates were maintained in a 37°C, 5% CO2 incubator for 12 days. To maintain a low-level of *PDIA4* and *P4HB* in the whole assay, siRNA transfection was repeated at the 6th day. After the 12 days of incubation, cell colonies were stained with crystal violet and photographed for further analysis.

### Bioinformatic Analysis

In this study, we developed a nomogram prediction model to predict the 1-, 3- and 5-year survival rate of glioma patients using package “rms” in R Language. PCA was performed to explore the transcriptomic differences within groups using R package “princomp” (David and Jacobs, 2014). In TCGA and CGGA datasets, the genes which were tightly correlated with the risk signature (Pearson correlation analysis, |R| > 0.5) were uploaded on DAVID website^5^. Then, Gene Ontology (GO) analysis and Kyoto Encyclopedia of Genes and Genomes (KEGG) pathway analysis were carried out to identified the biological processes tightly related to the risk signature. Gene-set enrichment analysis (GSEA) was applied to further identify biological functions of statistical difference between high-risk and low-risk groups by using the GSEA v3 software^6^ (Subramanian et al., 2005). CIBERSORT was also enrolled in this study to estimate the abundances of immune cells infiltrated in glioma tissue. In CIBERSORT analysis, a matrix of reference gene expression signatures (proposed by Newman et al.) was uploaded on the public website^7^ to estimate the relative proportions of each cell type (Newman et al., 2015, 2019).

### Statistical Analysis

The statistical analyses were mainly conducted using R language (version 3.6.2) and SPSS (version 16.0). Student’s *t*-test and One-way ANOVA were performed to compare differences among groups. The Kaplan-Meier (K-M) survival curves analysis was conducted to estimate survival distributions. Cox regression analysis, nomogram model and time ROC curve analysis were used to evaluate the prognostic value of the ER stress related signature (Robin et al., 2011). In this study, *p* < 0.05 was regarded as statistically significant.

## Results

### Cluster Model of ER Stress in Glioma Based on Consensus Clustering Analysis

In order to explore the association of ER stress status with the clinical characteristics and prognosis of patients with diffuse gliomas, consensus clustering analyses were performed with ER stress related genes in TCGA and CGGA RNA sequencing datasets. The results of consensus clustering suggested that patients could be grouped into two robust clusters and the optimal number of clusters (*k* = 2) was determined by cumulative distribution function (CDF) curves and consensus matrices (Supplementary Figures 1A,B). The heat map also showed that there were significant differences in the expression of ER stress related genes between these two clusters (Supplementary Figures 1C,D). Besides, we further explored the difference of clinical features between patients in different clusters through Chi-square test. As shown in Table 1, in TCGA dataset, patients in cluster 1 were mainly with younger age, lower grade of glioma, *IDH* mutated, *MGMT* promoter methylated, 1p/19q co-deleted and enriched in Neural and Proneural subtypes. Meanwhile, the clinicopathological characteristics of patients in cluster 2 were completely opposite. Consistent results were also obtained in CGGA dataset, with the exception of the MGMT Promoter status (Supplementary Table 1). Survival analysis suggested that the prognosis of patients in cluster 1 was much better than those in cluster 2 (Supplementary Figures 1E,F). Besides, considering the unique clinicopathological characteristics of IDH wildtype GBM samples, we also performed similar analysis in these samples. After dividing these samples into two clusters based on the expression level of ER stress related genes, the survival analysis show the prognosis of IDH wildtype GBM patients in two clusters tend to be different although the difference was not statistically significant in TCGA dataset (Supplementary Figures 1G,H, *P* = 0.13 and *P* = 0.03 for TCGA and CGGA dataset, respectively). In addition, the prognosis of IDH wildtype GBM patients is generally very poor, which leads to the classification and prognostic value of ER stress status were limited in these patients. In brief, these results indicated that the ER stress may participate in the malignant progression of glioma and have a significant impact on the prognosis of patients.

### Construction of ER Stress Related Signature in Diffuse Glioma

**FIGURE 1:**
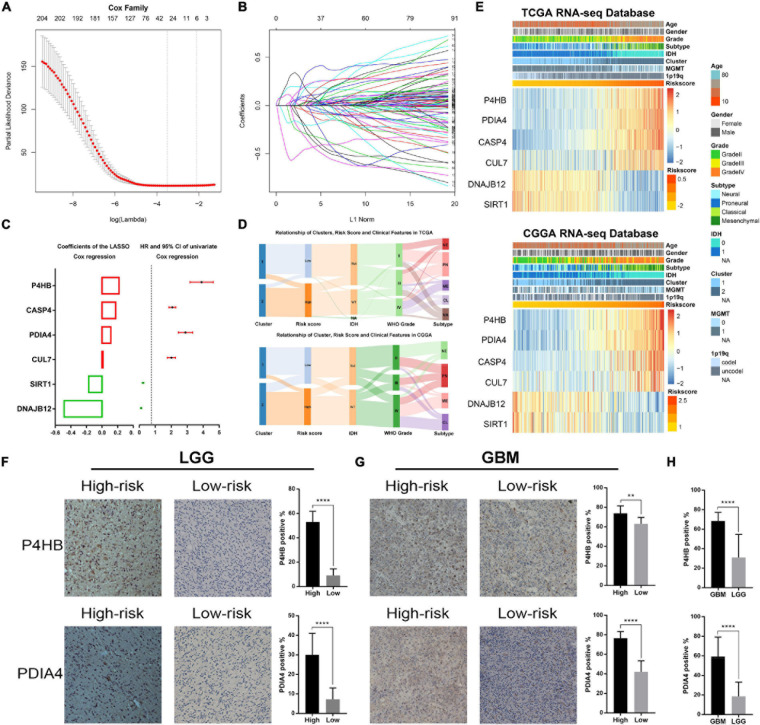
Identification of the ER stress-related signature. **(A)** Cross-validation for tuning parameter screening in the LASSO regression model. **(B)** The coefficient profiles of the LASSO regression model. **(C)** The six genes’ hazard ratios (HRs) and 95% confidence intervals (CIs) by univariate Cox regression analysis (right) and the coefficients by multivariate Cox regression analysis using LASSO (left). **(D)** Sankey diagram exhibiting the association among cluster, risk score and clinical features of glioma samples in TCGA and CGGA datasets. **(E)** Heat maps of six genes in the risk signature based on the risk score in TCGA and CGGA datasets. **(F)** IHC staining and positive rate statistics of *PDIA4* and *P4HB* in LGG samples from the CGGA dataset. **(G)** IHC staining and positive rate statistics of *PDIA4* and *P4HB* in GBM samples from the CGGA dataset. **(H)** Positive rate statistics of *PDIA4* and *P4HB* between LGG and GBM samples from the CGGA dataset. **p < 0.01, ****p < 0.0001.

Considering the prognostic value of ER stress in patients with gliomas, we proposed to construct a risk score model to evaluate the ER stress status of glioma samples more accurately. First, we performed univariate Cox regression analysis with the 256 ER stress related genes and screened out 208 genes that were significantly associated with the prognosis of glioma patients in TCGA dataset (*p* < 0.05). After that, these 205 genes were introduced into the LASSO Cox regression algorithm to select the most valuable predictive genes with non-zero regression coefficients and a six-gene risk signature was constructed (Figures 1A–C). In TCGA and CGGA datasets, the risk score of each patient was calculated by the formula “*Risk score*” mentioned in Materials and Methods section. We found that the clinical features of patients in the low-risk group and the high-risk group were highly consistent with those of cluster 1 and cluster 2, respectively (Figure 1D). Besides, the heatmaps exhibited an overview of association between ER stress-related genes and clinical characteristics, as the samples were ranked according to their risk scores (Figure 1E). Furthermore, we performed IHC staining of glioma samples from the CGGA dataset to evaluate the association of the expression level of ER stress biomarkers with risk score and WHO grade. Considering the significant difference of pathological characteristics between lower-grade glioma (LGG, WHO Grade II and III) and GBM, the IHC analysis was performed in LGG and GBM, respectively. The results suggested that the expression level of *PDIA4* and *P4HB* were higher in samples from high-risk group (Figures 1F,1G). Besides, we also found that the expression level of PDIA4 and P4HB in GBM samples was higher than that in LGG samples (Figure 1H).

### Association of the Risk Signature With Clinicopathologic Features and Genomic Alterations in Glioma

**FIGURE 2:**
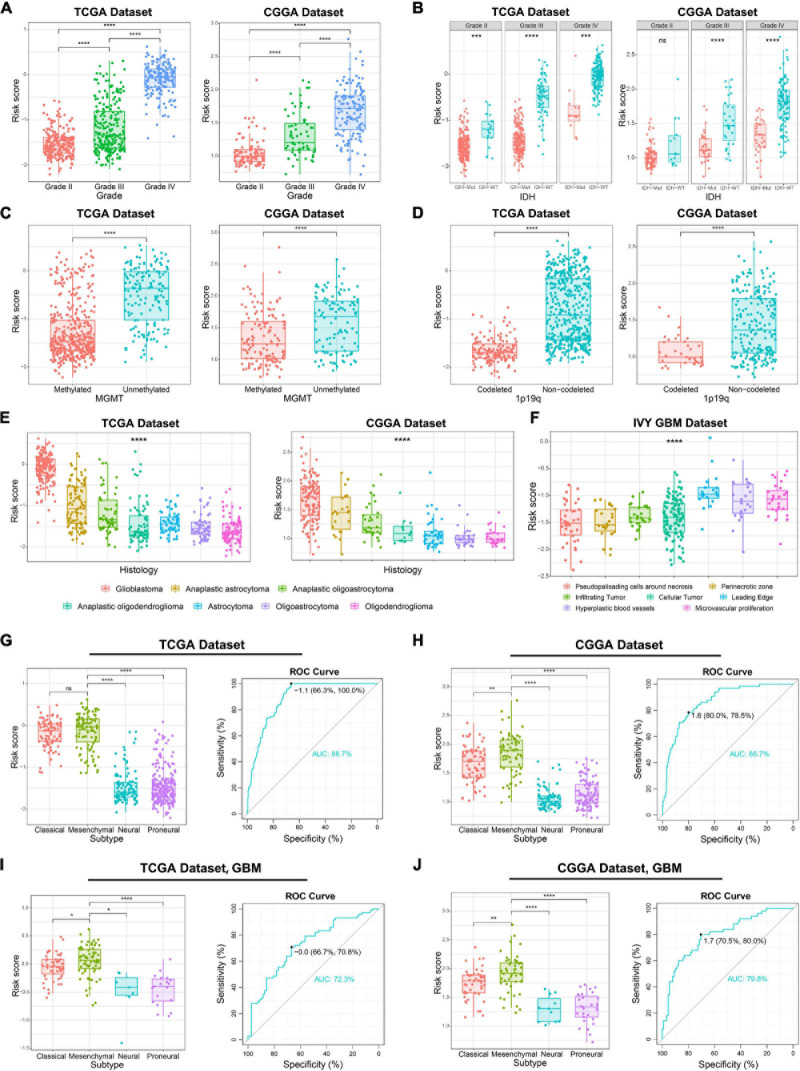
Association between the ER stress-related signature and clinicopathologic features in TCGA and CGGA datasets. **(A–E)** The association between risk score and WHO grade **(A)**, IDH mutation status **(B)**, *MGMT* promoter methylation **(C)**, 1p/19q co-deletion status **(D)** and histopathologic classification **(E)** of glioma patients. **(F)** The distribution of risk score in the different location of GBM in IVY GBM dataset. **(G,H)** In glioma samples, the Mesenchymal subtype had the highest risk score among four molecular subtypes. ROC curves predicted ER stress activation as a biomarker of Mesenchymal subtype glioma. **(I,J)** In GBM samples, the Mesenchymal subtype had the highest risk score among four molecular subtypes. ROC curves predicted ER stress activation as a biomarker of Mesenchymal subtype glioma. *p < 0.05, **p < 0.01, ***p < 0.001, ****p < 0.0001, ns: no statistically significant.

Considering the histopathological heterogeneity of glioma, we further investigate the association between risk score and clinicopathologic characteristics of patients in TCGA and CGGA datasets. We found that risk score was positively correlated with WHO grade of glioma (Figure 2A). Besides, we also found that the risk score of *IDH* wild-type glioma was higher than *IDH* mutation glioma (Figure 2B). The higher risk score was also detected in glioma samples with the molecular features of *MGMT* promoter unmethylated or 1p/19q non-codeleted (Figures 2C,D), which were generally thought to play a key role in the progression of glioma. Additionally, among various histopathologic types of glioma, GBM had the highest risk score (Figure 2E). Furthermore, we comprehensively analyzed RNA sequencing data for specific tumor anatomic structure in Ivy Glioblastoma Atlas Project dataset. The results suggested that glioma samples with high-risk score were mainly enriched in the leading edge, hyperplastic blood vessels and microvascular proliferation (Figure 2F), which are involved in the infiltration and proliferation of glioma. The four molecular subtypes of glioma defined by TCGA network have completely different molecular biological features. In this study, we found that the risk score was dramatically upregulated in gliomas of Mesenchymal subtype, which is generally associated with poor prognosis (Figures 2G,H). The ROC curve analysis also suggested that the risk signature could serve as a biomarker for the Mesenchymal subtype (Figures 2G,H). The association between the risk score and Mesenchymal subtype was also validated in the GBM samples (Figures 2I,J). In brief, these results indicated that ER stress might play an essential role in the malignant progression of gliomas.

**FIGURE 3:**
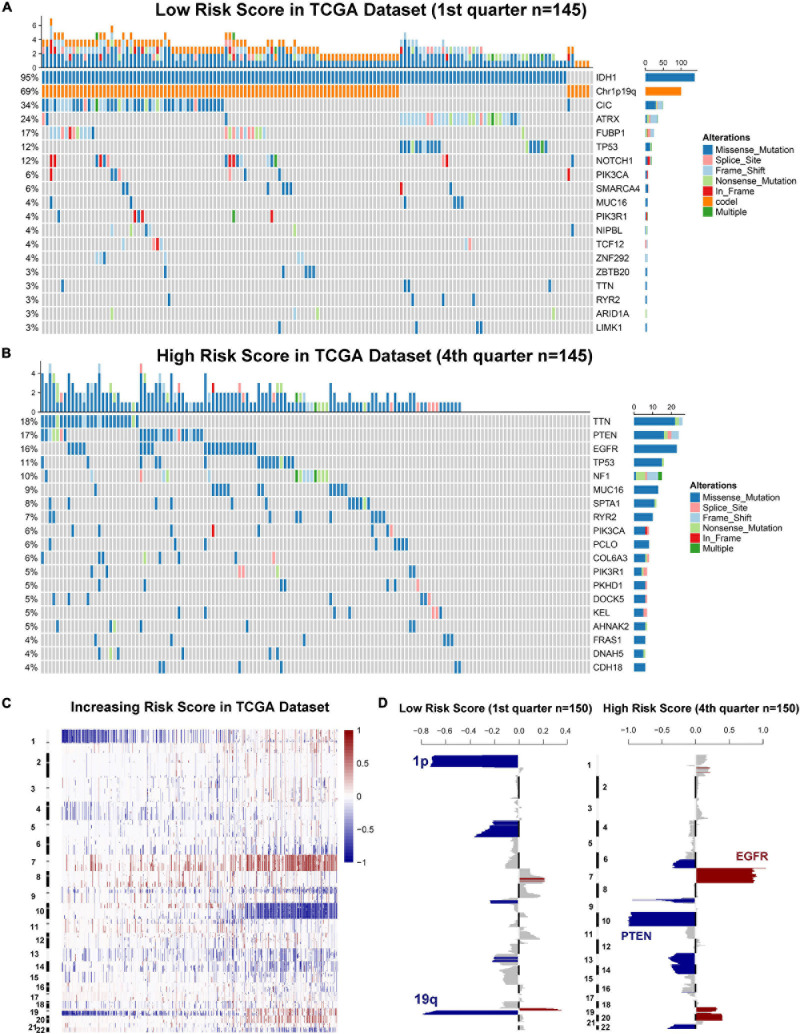
Gliomas with different ER stress activation status have distinct genomic profiles. **(A,B)** Distinct somatic mutations identified by comparing gliomas with low- and high-risk score. **(C)** CNV profiles with increasing order of the risk score of ER stress-related signature. **(D)** A distinct CNV spectrum defined by risk scores of ER stress-related signature. The amplification (red) and deletion (blue) of chromosome segment are presented.

Genomic instability including somatic mutations and copy number alterations (CNAs) drives the origin and development of gliomas. To further explore the molecular mechanisms influencing ER stress in gliomas, samples with available mutation and CNA information from TCGA dataset were analyzed. After dividing cases into four groups according to the order of increasing risk score. We found that higher incidence of 1p/19q codeletion and mutations in *IDH1*, *CIC*, *ATRX*, and *FUBP1* were observed in low-risk group (Figures 3A,B). Meanwhile, mutations in *EGFR*, *PTEN*, *NF1*, and *SPTA1* were mainly observed in high-risk group (Figures 3A,B). Besides, the results also showed that the mutations in *TTN*, *MUC16*, and *RYR2* tended to be different between low-risk and high-risk group. However, there was no significant difference in *TP53* mutation between patients stratified by risk score (Figures 3A,B). Furthermore, we also investigated the CNAs between cases in low-risk and high-risk group. The results suggested that 1p/19q codeletion, as a genomic hallmark of oligodendroglioma, tended to be enriched in low-risk group (Figures 3C,D). However, as a representative genomic alteration in GBM, chromosome 7 amplification accompanied chromosome 10 loss was mainly observed in the high-risk group (Figures 3C,D). Besides, it is worth mentioning that 7p11.2, which contains *EGFR* was one of the most commonly amplified genomic regions in high-risk group (Figure 3D). Meanwhile, the most frequently deleted region associated with high-risk score was 10q23.3, which contains the *PTEN* locus (Figure 3D).

### Prognostic Value of the ER Stress-Related Signature

**FIGURE 4:**
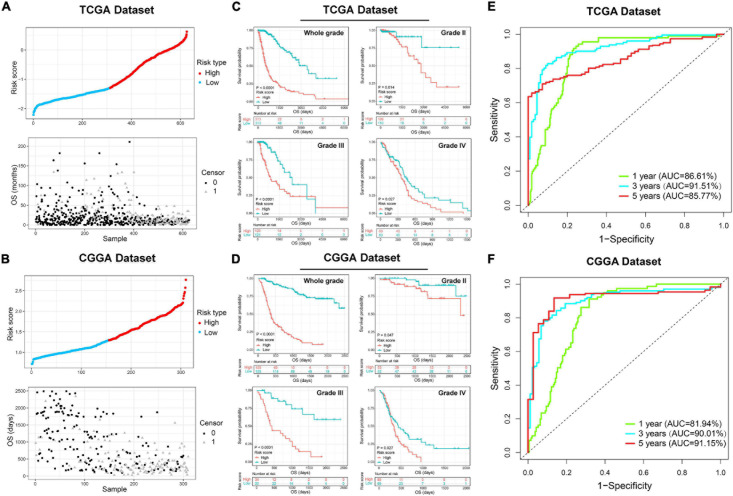
The prognostic value of the ER stress-related signature in TCGA and CGGA datasets. **(A,B)** The distribution of the risk score and survival overview of patients with gliomas. **(C,D)** K-M survival analyses of the risk signature in glioma patients stratified by WHO grade. **(E,F)** The time ROC curve analyses were performed to predict 1−, 3−, and 5-year OS according to risk score.

Since the ER stress-related signature was tightly associated with malignant progression of glioma, we further investigate the prognostic value of the risk signature. As the survival overview shown in Figures 4A,B, the prognosis of glioma patients deteriorated significantly as the risk score increases. The K-M survival analysis also suggested that patients in high-risk group experienced a shorter overall survival (OS) time in whole grade gliomas. Furthermore, similar results were also obtained in glioma patients of WHO grade II, WHO grade III and WHO grade IV in both CGGA and TCGA datasets (Figures 4C,D). K-M survival analyses were also performed in patients stratified by *IDH* mutant, *MGMT* promoter methylation or 1p/19q codeletion status. The results indicated that patients with higher risk score had inferior outcome in each subgroup (Supplementary Figure 2). In IDH-wild-type GBM patients, due to the limitation of sample size, the difference is not statistically significant, but it can still be found that patients in the low-risk group tend to have a better prognosis (Supplementary Figure 2). Besides, ROC curve analysis was performed to estimate the predictive value of risk signature for the survival rate. The results revealed that the risk signature had a favorable prognostic value with high time-dependent AUC for 1−, 3− and 5−year survival rate in both TCGA (86.61, 91.51, and 85.77%, respectively) and CGGA (81.94, 90.01, and 95.15%, respectively) datasets (Figures 4E,F). Considering that many clinicopathological factors have influence on the prognosis of glioma patients, Cox regression analysis was performed to further explore the clinical prognostic significance of the risk signature in gliomas. The univariate analysis showed that the risk score, age at diagnosis, WHO grade, TCGA subtypes, and the status of *IDH* mutation, *MGMT* promoter methylation and 1p/19q codeletion were significantly associated with OS in both TCGA and CGGA datasets (Table 2 and Supplementary Table 4). After adjusting these factors in the multivariate analysis, we found that the risk signature was still a significant survival predictor in both datasets. These results suggested that the ER-stress related signature may serve as an independent predictor for the poor prognosis of gliomas.

**TABLE 2:** Univariate and multivariate analysis of OS in TCGA sequencing dataset.

**Variables**	**Univariate analysis**		**Multivariate analysis**	
	**HR (95% CI)**	***p* value**	**HR (95% CI)**	***p* value**
Risk score	5.999 (4.754–7.570)	< 0.001	2.242 (1.310–3.839)	0.003
Age at Diagnosis	1.073 (1.061–1.085)	< 0.001	1.056 (1.040–1.073)	< 0.001
Gender	1.086 (0.818–1.440)	0.570	–	–
WHO Grade	4.862 (3.839–6.157)	< 0.001	1.553 (1.059–2.276)	0.024
TCGA Subtype	2.022 (1.779–2.299)	< 0.001	1.040 (0.823–1.313)	0.745
IDH mutation status	0.228 (0.158–0.329)	< 0.001	0.996 (0.474–2.091)	0.991
MGMT methylation	0.320 (0.234–0.439)	< 0.001	0.745 (0.496–1.118)	0.155
1p/19q co-deletion	0.227 (0.138–0.375)	< 0.001	0.628 (0.337–1.170)	0.143

In order to predict the survival rate of specific glioma patients more accurately, we constructed a nomogram model with the independent prognostic factors identified by multivariate COX analysis (Supplementary Figure 3A). Meanwhile, the calibration curves were performed to validate the accuracy of the nomogram model and the results showed a satisfactory concordance in the prediction of 1−, 3−, and 5-year OS in TCGA and CGGA datasets (Supplementary Figures 3B,C).

### Biological Functions Related to the 6-Gene Signature

**FIGURE 5:**
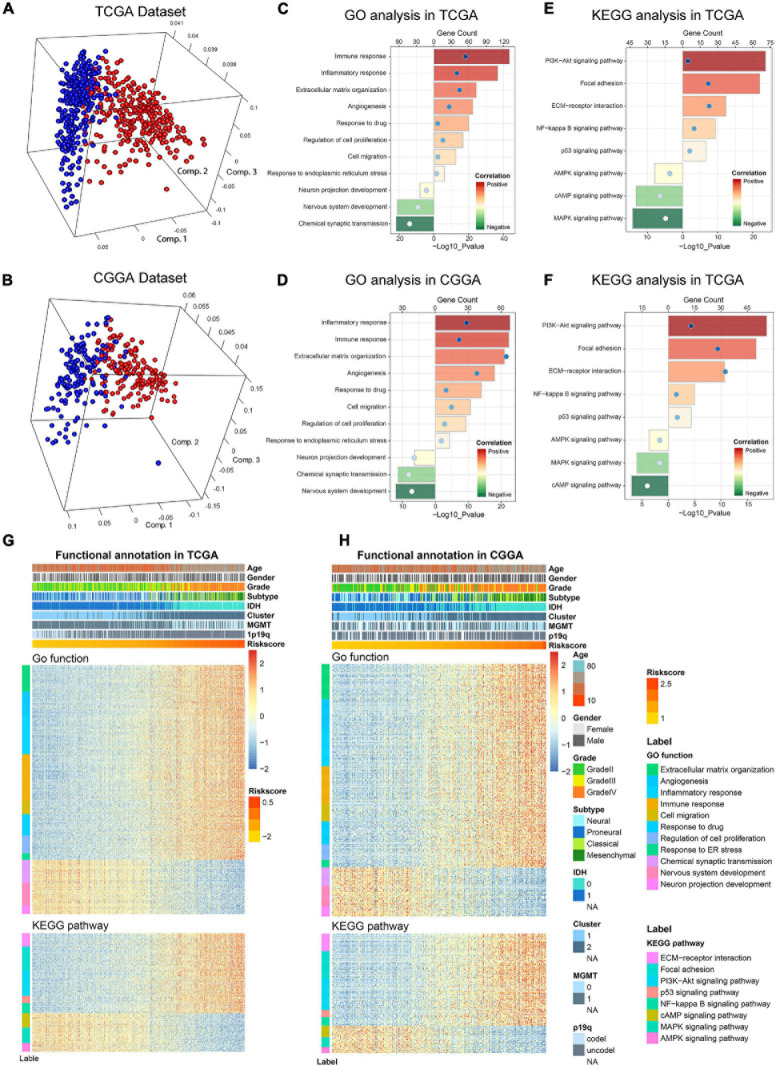
Biological functions and pathways analyses of the ER stress-related signature in TCGA and CGGA datasets. **(A,B)** There is a significant separation between high-risk and low-risk groups in PCA analysis based on whole gene expression data. **(C,D)** GO analyses were performed to explore the biological processes tightly related to the risk signature. **(E,F)** KEGG pathway analyses were performed to explore the signaling pathways tightly related to the risk signature. **(G,H)** The heatmaps showed the association among risk score, clinicopathological features and ER stress-related biological functions and pathways.

Considering the obvious difference in clinicopathological characteristics defined by distinct status of ER stress, principal components analysis (PCA) was performed based on the whole genome expression data to further explore the differences of gene expression pattern between patients in low− and high-risk group (Figures 5A,B). The results showed that cases in low− and high-risk group tended to distribute in different directions. To explore the biological functions associated with the risk signature in gliomas, A total of 4,975 and 2,572 genes which were tightly correlated with the risk signature were selected by performing Pearson correlation analysis (|R| > 0.5) in TCGA and CGGA datasets, respectively. Then, these genes were uploaded to DAVID website for GO analysis and KEGG pathway analysis. We found that the genes positively correlated with the risk score were mainly enriched in ER stress, inflammatory and immune response, cell migration, cell proliferation and angiogenesis in CGGA and TCGA datasets (Figures 5C,D). While the negatively related genes were involved in normal biological processes such as nervous system development, and others (Figures 5C,D). Besides, KEGG pathway analysis showed that the risk signature was positively related to *PI3K-AKT* and *NF-kappaB* signaling pathway, which were thought to be involved in the malignant progression of multiple cancers (Figures 5E,F). In addition, the heat maps displayed the expression profiles of biological processes related to the risk signature in TCGA and CGGA datasets (Figures 5G,H). Meanwhile, we also performed GSEA to verify aforementioned results and obtained similar conclusions (Supplementary Figure 4). These findings suggested that the risk score was positively corelated to the malignant biological functions and signaling pathways in gliomas.

### Association Between the Risk Signature and Immunologic Events

**FIGURE 6:**
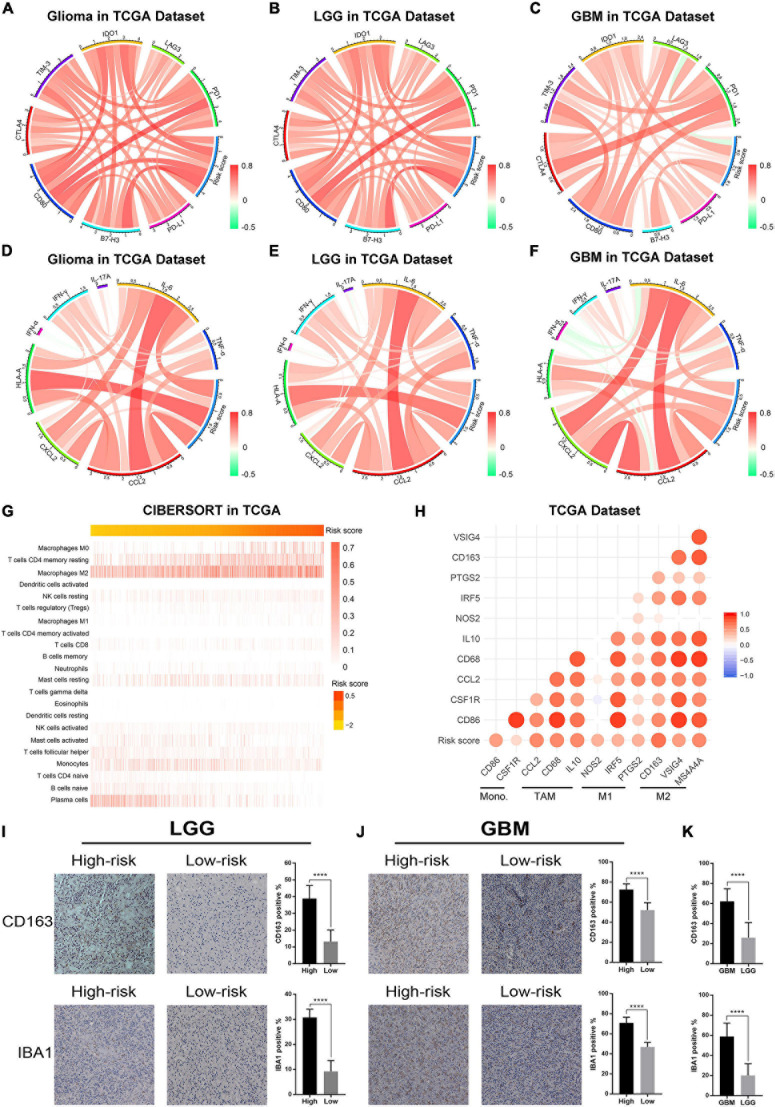
The ER stress activation related immunologic events in glioma samples in TCGA dataset. **(A–C)** Correlation analysis between risk score and immune checkpoints in whole gliomas, low-grade gliomas and GBM, respectively. **(D–F)** Correlation analysis between risk score and inflammatory genes in whole gliomas, low-grade gliomas and GBM, respectively. **(G)** CIBERSORT was performed to evaluate the proportion of various types of immune cell infiltrated into glioma samples. **(H)** Correlation analysis between risk score and biomarkers of monocytes and macrophages. **(I)** IHC staining and positive rate statistics of *CD163* and *IBA1* in LGG samples from the CGGA dataset. **(J)** IHC staining and positive rate statistics of *CD163* and *IBA1* in GBM samples from the CGGA dataset. **(K)** Positive rate statistics of *CD163* and *IBA1* between LGG and GBM samples from the CGGA dataset. ****p < 0.0001.

Previous studies reported that ER stress played an essential role in immune and inflammation response of multiple diseases (Engin, 2016; Feng et al., 2017; Gerakis and Hetz, 2018). This opinion was confirmed in glioma by our study. To further investigate the relationship between ER stress and immune response, several genes of immune checkpoints, such as *B7-H3*, *CTLA-4*, *TIM-3*, *PD-1*, and others were enrolled in this study. The results demonstrated that the risk signature was positively correlated to most of the immune checkpoints in CGGA and TCGA datasets, especially *B7-H3* (Figure 6A and Supplementary Figure 5A). It means that the excessive upregulation of ER stress may inhibit anti-tumor immune response in gliomas (Wang et al., 2016; Li et al., 2017). Besides, these results were also verified in lower-grade glioma (LGG) and GBM samples in two datasets (Figures 6B,C and Supplementary Figures 5B,C). In addition, we also selected several representative inflammatory genes to further investigate the association between ER stress and inflammation response. We found that the risk signature was positively correlated with *IL-6*, *CCL2*, and *HLA-A* (Figures 6D–F and Supplementary Figures 5B,C), suggesting T lymphocyte and macrophages mediated inflammatory response were involved in samples from high-risk group (Kolls and Linden, 2004; Germano et al., 2008). Considering that immune cell infiltration is an important component of tumor immune microenvironment (Gajewski et al., 2013; Gabrusiewicz et al., 2016), we further evaluated the proportion of various types of immune cell with CIBERSORT for CGGA and TCGA datasets. As samples were sorted by risk score in increasing order, the abundance of macrophages in M0 and M2 phase which had been reported to play an immunosuppressive role in cancers increased (Gabrusiewicz et al., 2016; Figure 6G and Supplementary Figure 5G). To verify these results, we also enrolled biomarkers of macrophages in different phases and found that the risk signature was significantly correlated to tumor-associated macrophage (TAM) and macrophages in M2 (Figure 6H and Supplementary Figure 5H). Besides, IHC staining was performed to further validate the infiltration of macrophages in tumor tissue from glioma patients stratified by risk score in CGGA dataset. We found that the expression levels of *CD163* and *IBA1* significantly increased in LGG and GBM samples from high-risk group, suggesting that the activation of ER stress could promote the infiltration of macrophages in M2 phase (Figures 6I,J). Besides, the results also suggested that *CD163* and *IBA1* were overexpressed in GBM (Figure 6K). These findings might provide a new insight into the suppression of anti-tumor immune response in gliomas.

### ER Stress Inhibition Attenuated Glioma Progression *in vitro*

**FIGURE 7:**
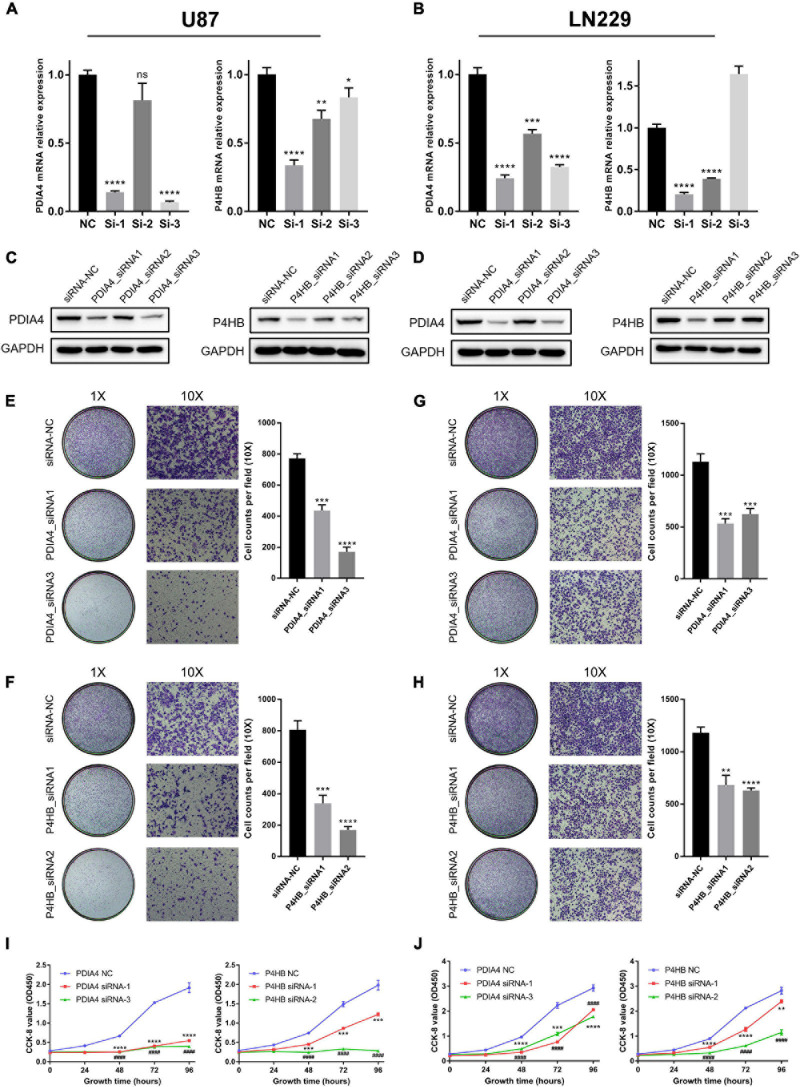
ER stress activation promotes migration and proliferative capacity of glioma cell lines *in vitro*. **(A,B)** The real-time quantitative PCR (qPCR) assay of *PDIA4* and *P4HB* mRNA expression in LN229 and U87 cell lines after applying specific siRNA and negative control. **(C,D)** The western blot analysis of *PDIA4* and *P4HB* protein expression in LN229 **(C)** and U87 **(D)** cell lines after applying specific siRNA and negative control. **(E–H)** Transwell migration assay of LN229 **(E,F)** and U87 **(G,H)** cell lines treated with specific siRNA and negative control. **(I,J)** CCK-8 assay displaying proliferation of LN229 **(I)** and U87 **(J)** cell lines treated with specific siRNA and negative control. *p < 0.05, **p < 0.01, ***p < 0.001, ****p < 0.0001.

In this study, we performed experiments *in vitro* to further validate the results of bioinformatics analyses. First, LN229 and U87 cell lines were transfected with specific siRNA to knockdown the expression level of *P4HB* and *PDIA4*, which were considered as important biomarkers of ER stress activation (Joo et al., 2007; Kranz et al., 2017; Winship et al., 2017; Liu et al., 2019; Wang et al., 2020). WB and qPCR assays showed that both of the mRNA and protein expressions of *PDIA4* and *P4HB* could be significantly downregulated by specific siRNA (Figures 7A–D). Then, the transwell migration assays and cell scratch assays suggested that *PDIA4* or *P4HB* knockdown could significantly suppress cell migration of LN229 and U87 cells *in vitro* (Figures 7E–H and Supplementary Figures 7A,B). Besides, CCK-8 and clonogenic assays indicated that proliferative capacity and clonogenicity of glioma cell lines were attenuated after transfection with *PDIA4* or *P4HB* siRNA (Figures 7I,J and Supplementary Figures 7C,D). These results suggested that the activation of ER stress played an essential role in malignant progression of gliomas. Meanwhile, it is also indicated that anti-*PDIA4* and/or anti-*P4HB* therapy may be a novel approach for glioma treatments.

## Discussion

As the most malignant primary neoplasms in the central nervous system, the pathogenesis and progression of gliomas is a multi-factor and multi-step process (Ricard et al., 2012; Gusyatiner and Hegi, 2018). Due to the high invasiveness and heterogeneity of glioma cells, the efficacy of traditional treatment strategies was limited and the prognosis of glioma patients was dismal (Jiang et al., 2016; Reifenberger et al., 2017). Thus, novel therapeutic approaches and targets are urgently needed. In the past decade, ER stress becomes an increasingly eye-catching research field in various human cancers, which brings new hope for the improvement of individualized treatment of gliomas.

Endoplasmic reticulum is the most important organelle for protein synthesis and intracellular calcium storages, which is also involved in the regulation of multiple cellular signaling pathways (Tsai and Weissman, 2010; Schwarz and Blower, 2016). Upon the induction of intracellular and extracellular stress, the accumulation of unfolded or misfolded proteins can activate the unfolded protein response (UPR) to restore the homeostasis of ER (Namba, 2015). The *PERK/ATF4/CHOP*, *IRE-1/XBP1*, and *ATF6* signaling pathways have an essential role in regulating ER stress-induced physiological responses in cells (Lee, 2005; Luo and Lee, 2013). However, excessive and durable activation of ER stress can also induce cell apoptosis (Szegezdi et al., 2006). ER stress is involved in the process of many diseases, such as Parkinson’s disease (PD), Alzheimer’s disease (AD), diabetes and tumors (Engin, 2016; Mercado et al., 2016; Gerakis and Hetz, 2018). The in-depth study of ER stress provides an important reference for the clinical treatment of these diseases.

In malignancy tissues, cancer cells which are constantly exposed to hypoxic tumor microenvironment and intracellular DNA damage stress have a high risk of ER stress activation. These mechanisms have been proved to participate in the malignant progression and drug resistance of many human cancers (Dasari and Tchounwou, 2014). The branch of *PERK-ATF4* can activate the epithelial-to-mesenchymal transition (EMT) response and promote the metastasis of breast cancer cells (Feng et al., 2017). In hepatocellular and prostate cancer, the transcription factors from UPR can bind and activate the promoter of *VEGF*, which resulting in the proliferation and migration of endothelial cells (Pereira et al., 2014; Ramakrishnan et al., 2014). Besides, the activation of ER stress can also induce the dormancy of cancer cells. Cellular dormancy can block the division and proliferation of cancer cells by arresting them in the G0/G1 phase of cell cycle (Aguirre-Ghiso, 2007; Vera-Ramirez and Hunter, 2017). The cancer cells which survive in a quiescent state are not sensitive to radiotherapy or chemotherapy, which eventually lead to tumor recurrence and poor prognosis of patients. Furthermore, relevant studies have proved that *IRE-1-XBP1* signaling pathway could inhibit anti-tumor immune responses and provide an opportunity for the formation of tumors (Cubillos-Ruiz et al., 2015). These results suggested that ER stress plays a key role in the remodeling of tumor immune microenvironment and h serving as a novel target of immunological therapy. Although serval studies have confirmed the therapeutic value of ER stress in glioma, the biological functions of ER stress in glioma have not been completely understood, especially the role of ER stress in the regulation tumor immune microenvironment (Chang et al., 2020).

In the present study, we constructed a risk signature to evaluate the ER stress activation of glioma samples from TCGA and CGGA datasets. Our findings showed that ER stress activation was tightly correlated to the malignant clinicopathological features and genomic alterations. These results revealed the significance of ER stress and the molecular mechanisms influencing ER stress activation in gliomas. It was interesting to note that the low-risk score was tightly associated with 1p/19q codeletion, suggesting the risk signature may server as a marker of oligodendrogliomas. Besides, K-M survival analysis and COX regression analysis suggested that this risk signature was an independent prognostic factor of patients with gliomas. Furthermore, both bioinformatics analysis and *in vitro* experiments indicated that ER stress activation can significantly promote the proliferation and migration of glioma cells. Besides, we also found that ER stress was tightly associated with the regulation of tumor immune microenvironment in glioma, especially the infiltration of macrophages in M2 phase. To further validated the results of bioinformatics analysis, IHC analysis and cell biology experiments were performed. The results suggested that ER stress activation promoted the malignant progression of glioma.

According to the previous researches, protein disulfide isomerase (PDI) family members play an essential role in ER stress activation (Hsu et al., 2019; Zhang et al., 2019). The *P4HB* (also known as *PDIA1*) and *PDIA4*, which were chosen from the six-gene signature, belong to the protein PDI protein family and have been proved to be related to ER stress activation in human cancers (Joo et al., 2007; Kranz et al., 2017; Winship et al., 2017; Liu et al., 2019; Wang et al., 2020), especially the PERK signaling pathway (Kranz et al., 2017, 2020). Serval recent researches has pointed out that *P4HB* and *PDIA4* correlated with the malignant progression of glioma, but these conclusions have not been sufficiently verified by *in vitro* experiments (Zou et al., 2018; Li et al., 2021). Besides, the *P4HB* and *PDIA4*, which have significant prognostic value in gliomas, were also the important components of the ER stress-related signature. Thus, in *in vitro* experiments, *P4HB* and *PDIA4* were selected as biomarkers of ER stress activation. We found that knockdown of the *P4HB* and *PDIA4* could significantly reduce the proliferation and migration of glioma cell lines *in vitro*. These findings indicated that *P4HB* and *PDIA4* may serve as promising target for glioma therapy. However, we also recognized that the current evidences were limited and future studies are necessary. Other four genes in the risk signature (*CASP4*, *CUL7*, *DNAJB12*, and *SIRT1*) were not directly participated in the three classic ER stress signaling pathway mentioned before. According to the findings in this study, we hypothesized that the other four genes may be involved in the regulation of ER stress activation through unknown molecular mechanisms, which is valuable for us to further investigate and discuss.

In this study, we have confirmed the importance of ER stress activation in the progression of glioma through sufficient bioinformatics analysis and *in vitro* experiments. Although, the prognostic value and biological functions of ER-stress in gliomas were fully discussed, the specific molecular mechanism underlying the interaction between ER stress and tumor immune microenvironment remains unclear. Thus, more in-depth exploration is needed. Taken together, our findings revealed the functional roles of ER stress in glioma and provided promising targets to enhance the individualized treatment of patients with glioma.

## Conclusion

In this study, we constructed a risk signature to evaluated the ER stress activation status and prognosis of glioma patients. Functional analyses suggested that the ER stress activation was tightly correlated with cell migration and cell proliferation gliomas, which were further verified in the experiments *in vitro*. Besides, we also found that ER stress activation could promote the infiltration of macrophages in M2. *P4HB* and *PDIA4*, which played an essential role in the activation of ER stress, may serve as a novel potential target to enhance the anticancer therapies. Accordingly, these findings provided an important reference in personalized clinical treatment for glioma patients.

## Data Availability Statement

The datasets presented in this study can be found in online repositories. The names of the repository/repositories and accession number(s) can be found in the article/Supplementary Material.

## Ethics Statement

The studies involving human participants were reviewed and approved by Institutional Review Board (IRB) of Beijing Tiantan Hospital Affiliated to Capital Medical University. The patients/participants provided their written informed consent to participate in this study.

## Author Contributions

RH, GL, and TJ: conception and design. RH, ZW, and HH: development of methodology. RH, GL, and KW: acquisition of data. RH, KW, and FZ: analysis and interpretation of data. RH and GL: writing, review and/or revision of the manuscript. TJ and HH: administrative, technical, or material support. TJ: study supervision. All authors read and approved the final manuscript.

## Conflict of Interest

The authors declare that the research was conducted in the absence of any commercial or financial relationships that could be construed as a potential conflict of interest.

## Footnotes

1
http://cancergenome.nih.gov/

2
http://www.cgga.org.cn

3
http://glioblastoma.alleninstitute.org/

4
http://www.broad.mit.edu/gsea/msigdb/

5
http://david.ncifcrf.gov/

6
http://www.broadinstitute.org/gsea/index.jsp

7
https://cibersort.stanford.edu/

## Supplementary Material

Supplementary Figure 1 Consensus clustering for ER stress-related genes in glioma patients from TCGA and CGGA dataset. **(A)** Consensus clustering CDF for *k* = 2 to *k* = 10. **(B)** Consensus clustering matrix of glioma samples from TCGA and CGGA dataset for *k* = 2, respectively. **(C,D)** Heatmap of two clusters identified by the top 50 variable expression genes. **(E,F)** K-M survival curve analyses of patients in two clusters. **(G,H)** By performing Consensus clustering analysis, IDH wildtype GBM patients were also divided into two clusters, K-M survival curve analyses was performed to evaluated the prognosis of IDH wildtype GBM patients in two clusters.

Supplementary Figure 2 K-M survival analysis of the risk signature in glioma patients stratified by *IDH* mutation status, *MGMT* promoter methylation and 1p/19q co-deletion status in TCGA and CGGA datasets.

Supplementary Figure 3 A nomogram model for predicting overall survival of patients with glioma. **(A)** A nomogram model was constructed by integrating the clinicopathologic features and risk score of patients in the TCGA dataset. **(B,C)** The calibration curve of nomogram for predicting OS at 1-, 3- and 5-years in the TCGA and CGGA datasets.

Supplementary Figure 4 GSEA analysis was performed to investigate the biological functions and pathways that were tightly correlated with the ER stress activation.

Supplementary Figure 5 The ER stress activation related immunologic events in glioma samples in CGGA dataset. **(A–C)** Correlation analysis between risk score signature and immune checkpoints in whole gliomas, low-grade gliomas and GBM, respectively. **(D–F)** Correlation analysis between risk score and inflammatory genes in whole grade of gliomas, low-grade gliomas and GBM, respectively. **(G)** CIBERSORT evaluating the proportion of various types of immune cell infiltrated into glioma samples. **(H)** Correlation analysis between risk score and biomarkers of monocytes and macrophages.

Supplementary Figure 6 K-M survival analysis of each independent gene in the ER stress-related signature in TCGA and CGGA datasets.

Supplementary Figure 7 ER stress activation promotes migration and proliferative capacity of glioma cell lines *in vitro*. **(A,B)** Cell scratch assay of U87 and LN229 cell lines treated with *PDIA4* siRNA, *P4HB* siRNA and negative control. **(C,D)** Clonogenic assay of U87 and LN229 cell lines treated with *PDIA4* siRNA, *P4HB* siRNA and negative control.

Supplementary Table 1 Characteristics of patients in cluster 1 and cluster 2 in CGGA databset.

Supplementary Table 2 ER stress related gene list.

Supplementary Table 3 Univariate Cox analysis of ER stress-related genes.

Supplementary Table 4 Univariate and multivariate analysis of OS in CGGA sequencing dataset.
