# Corrigendum: Comprehensive analysis of the clinical and biological significances of endoplasmic reticulum stress in diffuse gliomas

**DOI:** 10.3389/fcell.2022.905911

**Published:** 2022-09-21

**Authors:** Ruoyu Huang, Guanzhang Li, Kuanyu Wang, Zhiliang Wang, Fan Zeng, Huimin Hu, Tao Jiang

**Affiliations:** ^1^ Department of Molecular Neuropathology, Beijing Neurosurgical Institute, Capital Medical University, Beijing, China; ^2^ Department of Neurosurgery, Beijing Tiantan Hospital, Capital Medical University, Beijing, China; ^3^ Chinese Glioma Cooperative Group (CGCG), Beijing, China; ^4^ Department of Gamma Knife Center, Beijing Neurosurgical Institute, Capital Medical University, Beijing, China

**Keywords:** glioma, ER stress, signature, prognosis, tumor immune environment

In the original article, there was a mistake in **Supplementary Figure S7** as published. In Supplementary Figure S7B (PDIA4_siRNA3, 48 h), the wrong image was inserted.

The authors apologize for this error and state that this does not change the scientific conclusions of the article in any way. The original article has been updated.

