# Supplementary figures and images for: Comprehensive Analysis of the Clinical and Biological Significances of Endoplasmic Reticulum Stress in Diffuse Gliomas
Source: Front Cell Dev Biol. 2021 Jul 9;9:619396. doi: 10.3389/fcell.2021.619396 (PMC8301220; doi:10.3389/fcell.2021.619396)

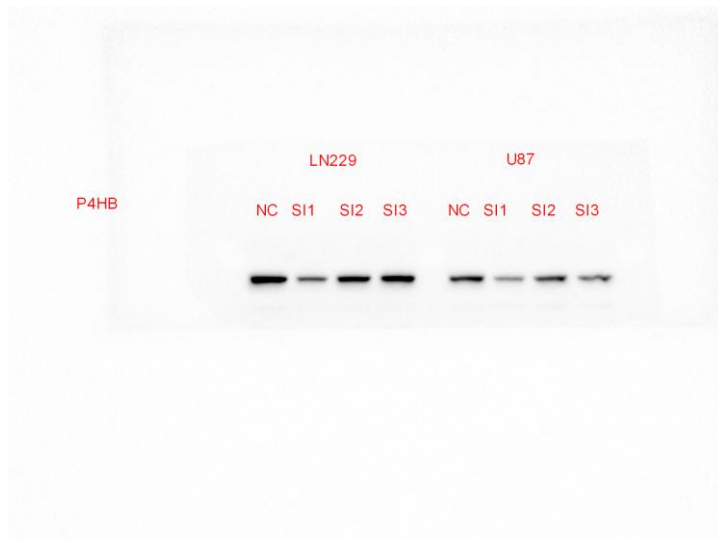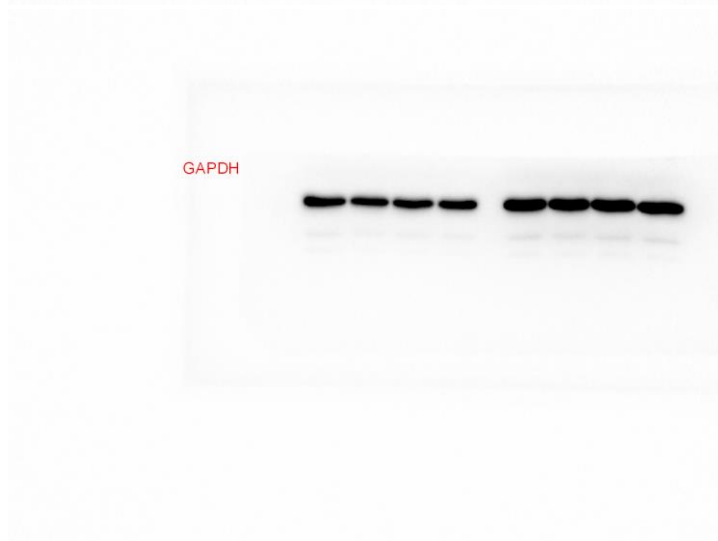

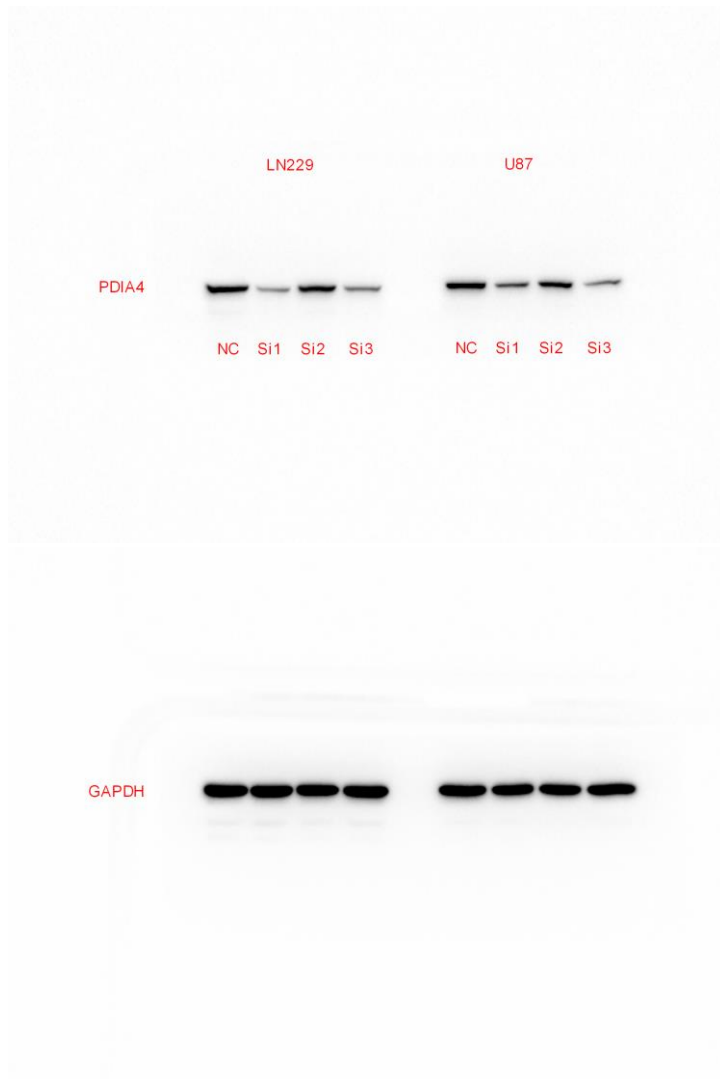

Supplement: Supplementary file 1 [file Data_Sheet_1.PDF]
