# Supplementary material for: Comprehensive Analysis of the Clinical and Biological Significances of Endoplasmic Reticulum Stress in Diffuse Gliomas
Source: Front Cell Dev Biol. 2021 Jul 9;9:619396. doi: 10.3389/fcell.2021.619396 (PMC8301220; doi:10.3389/fcell.2021.619396)
Supplement: Supplementary Figure 1 — Consensus clustering for ER stress-related genes in glioma patients from TCGA and CGGA dataset. (A) Consensus clustering CDF for k = 2 to k = 10. (B) Consensus clustering matrix of glioma samples from TCGA and CGGA dataset for k = 2, respectively. (C,D) Heatmap of two clusters identified by the top 50 variable expression genes. (E,F) K-M survival curve analyses of patients in two clusters. (G,H) By performing Consensus clustering analysis, IDH wildtype GBM patients were also divided into two clusters, K-M survival curve analyses was performed to evaluated the prognosis of IDH wildtype GBM patients in two clusters. [file Data_Sheet_2.pdf]

Supplementary materials

Supplementary Figures

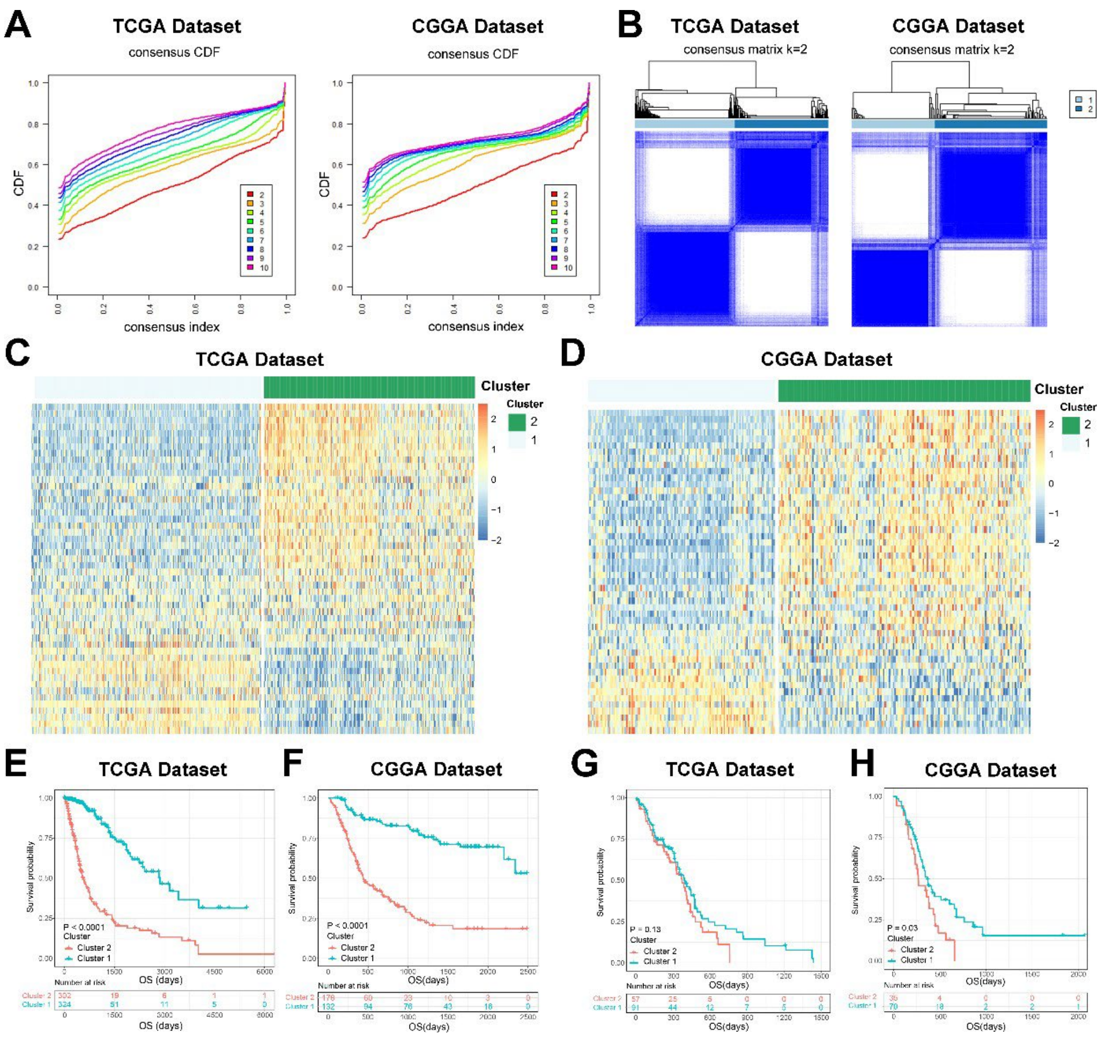

Supplementary Fig. 1. Consensus clustering for ER stress-related genes in glioma patients from TCGA and CGGA dataset.

(A) Consensus clustering CDF for k = 2 to k = 10. (B) Consensus clustering matrix of glioma samples from TCGA and CGGA dataset for k = 2, respectively. (C, D) Heatmap of two clusters identified by the top 50 variable expression genes. (E, F) K-M survival curve analyses of patients in two clusters. (G, H) By performing Consensus clustering analysis, IDH wildtype GBM patients were also divided into two clusters, K-M survival curve analyses was performed to evaluated the prognosis of IDH wildtype GBM patients in two clusters.

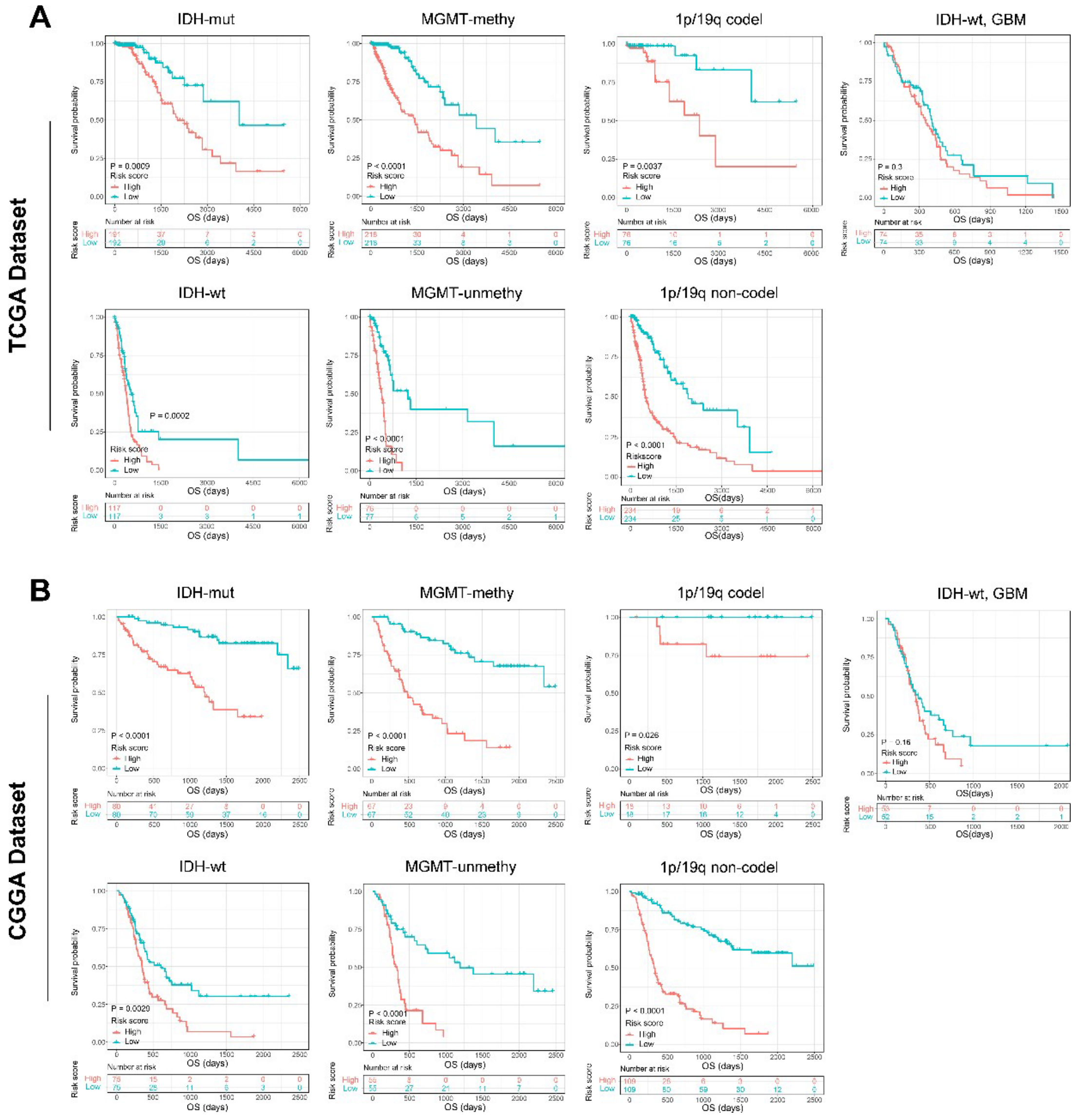

Supplementary Fig. 2. K-M survival analysis of the risk signature in glioma patients stratified by IDH mutation status, MGMT promoter methylation and 1p/19q co-deletion status in TCGA (A) and CGGA (B) datasets.

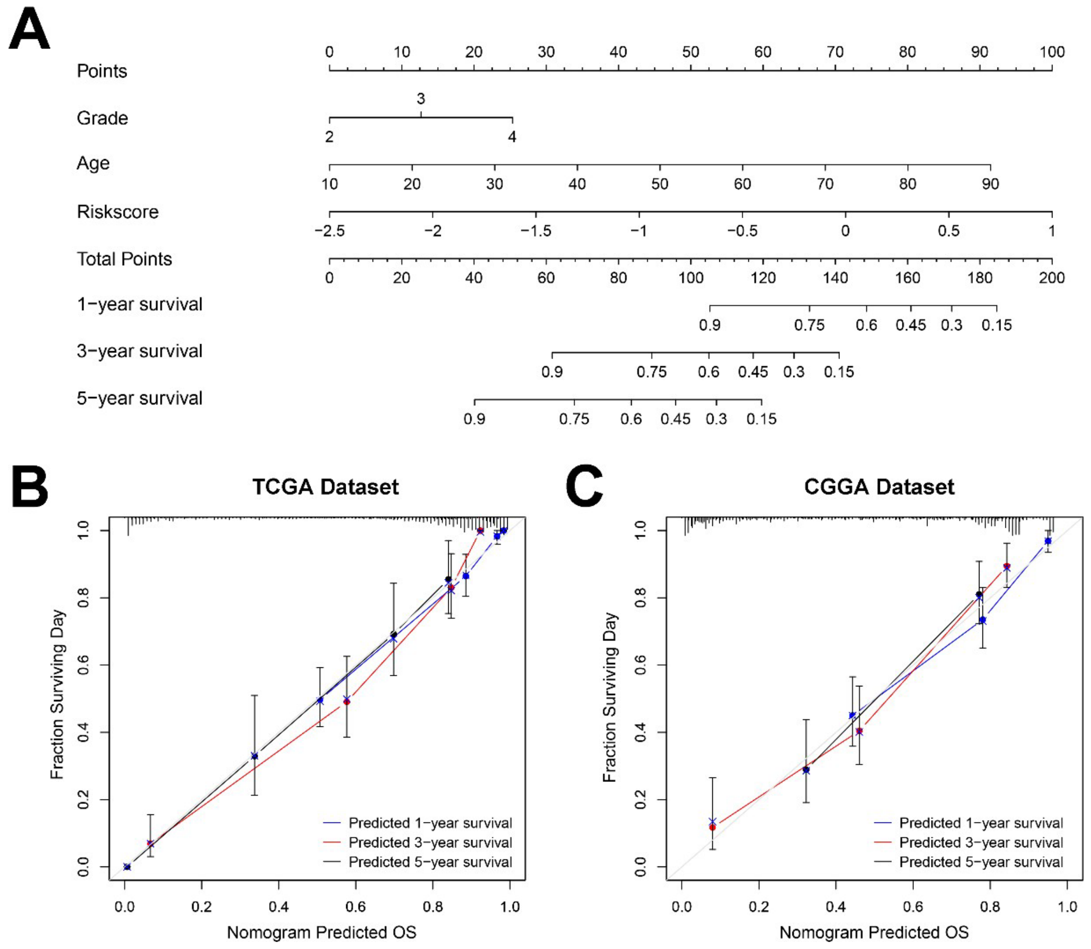

Supplementary Fig. 3. A nomogram model for predicting overall survival of patients with glioma.

(A) A nomogram model was constructed by integrating the clinicopathologic features and risk score of patients in the TCGA dataset. (B, C) The calibration curve of nomogram for predicting OS at 1-, 3- and 5-years in the TCGA and CGGA datasets.

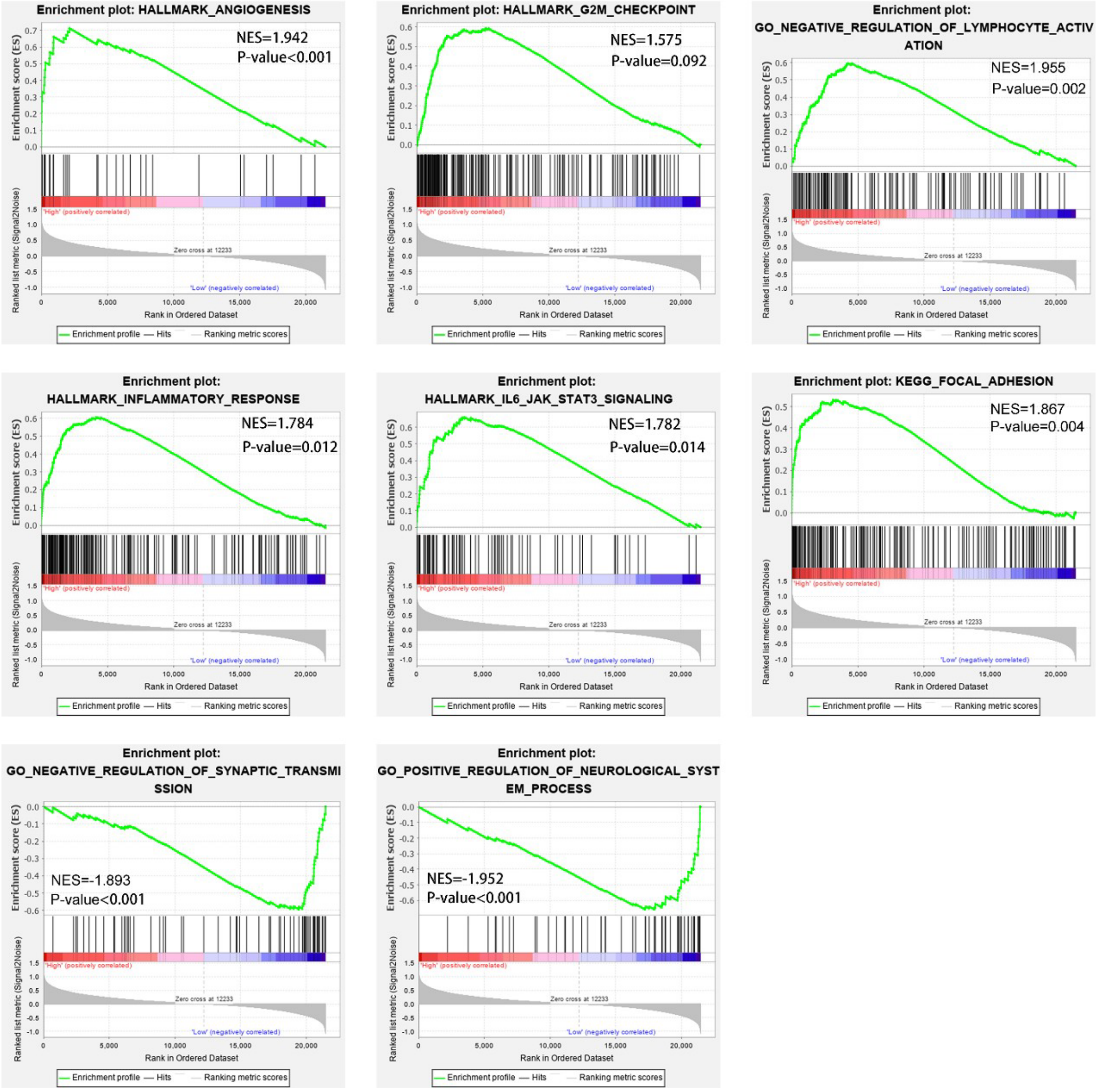

Supplementary Fig. 4. GSEA analysis was performed to investigate the biological functions and pathways that were tightly correlated with the ER stress activation.

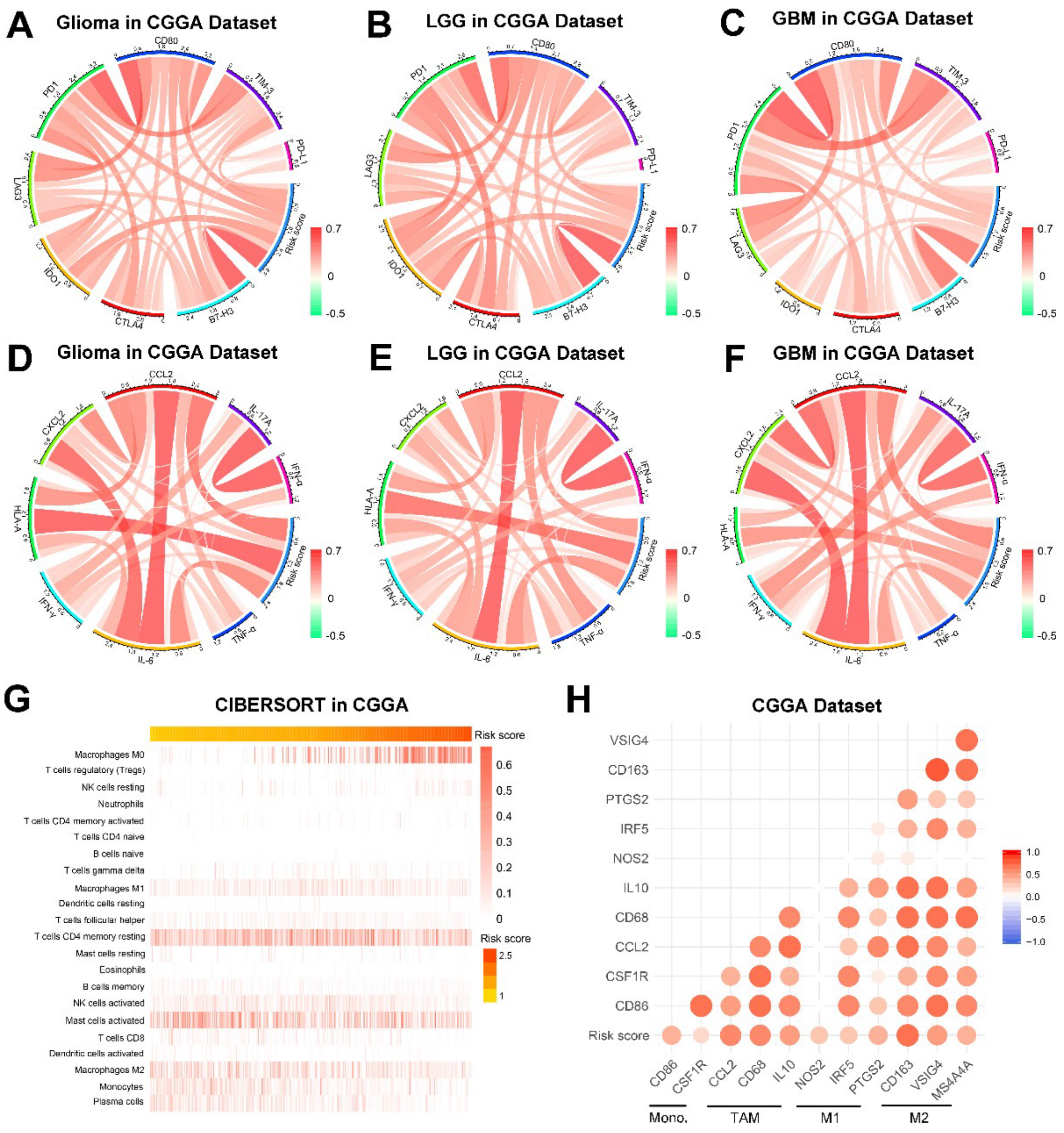

Supplementary Fig. 5. The ER stress activation related immunologic events in glioma samples in CGGA dataset.

(A-C) Correlation analysis between risk score signature and immune checkpoints in whole gliomas, low-grade gliomas and GBM, respectively. (D-F) Correlation analysis between risk score and inflammatory genes in whole grade of gliomas, low-grade gliomas and GBM, respectively. (G) CIBERSORT evaluating the proportion of various types of immune cell infiltrated into glioma samples. (H) Correlation analysis between risk score and biomarkers of monocytes and macrophages.

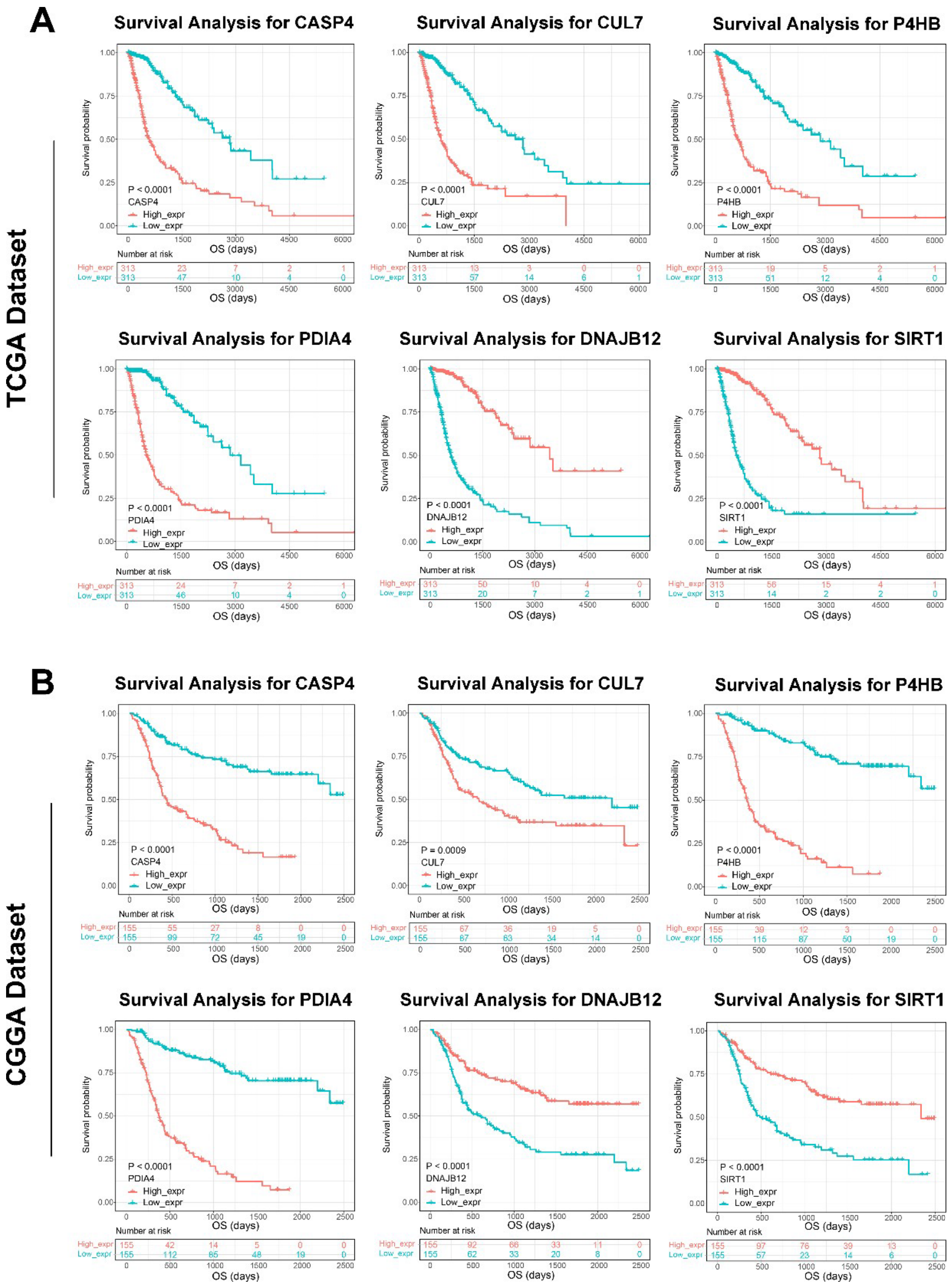

Supplementary Fig. 6. K-M survival analysis of each independent gene in the ER stress-related signature in TCGA (A) and CGGA (B) datasets.

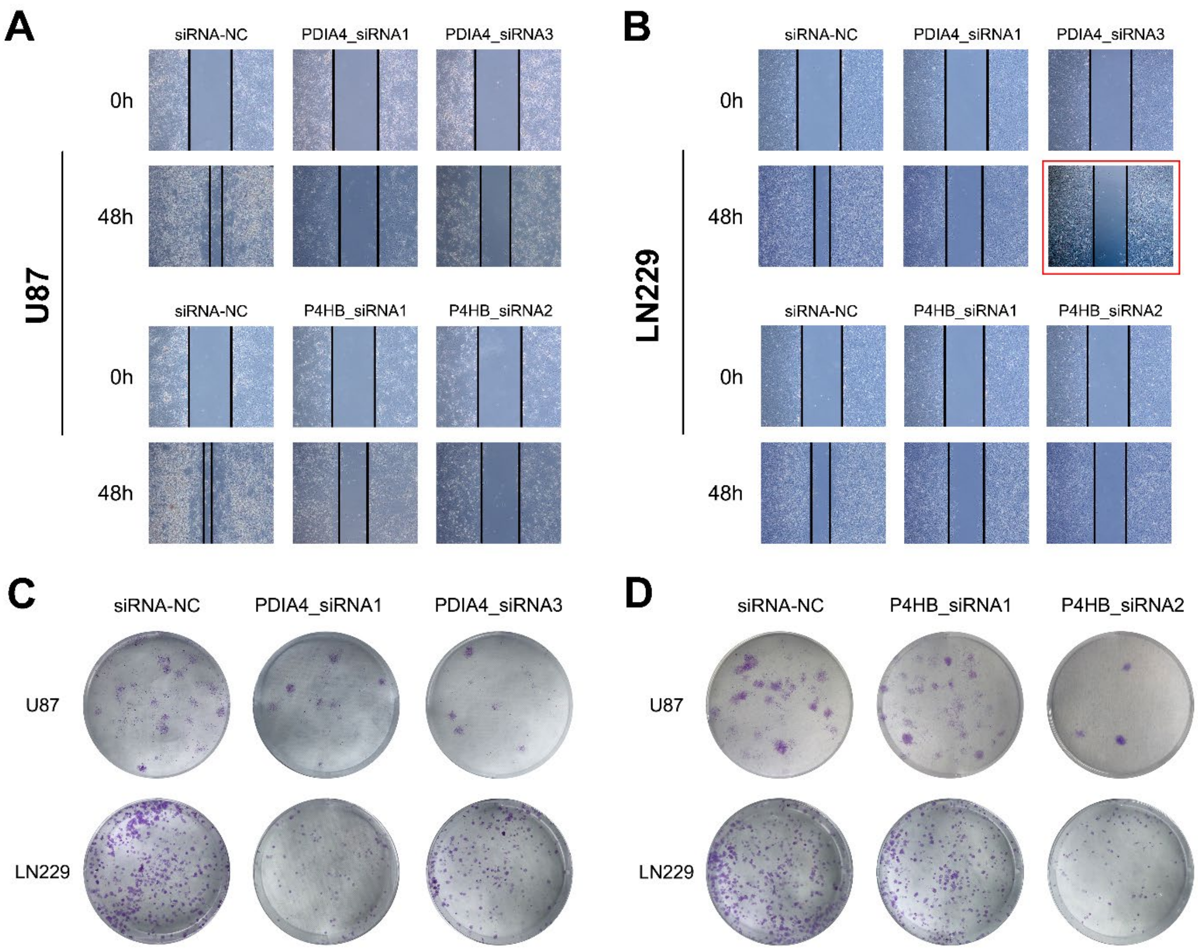

Supplementary Fig. 7. ER stress activation promotes migration and proliferative capacity of glioma cell lines *in vitro*.

(A, B) Cell scratch assay of U87 and LN229 cell lines treated with PDIA4 siRNA, P4HB siRNA and negative control. (C, D) Clonogenic assay of U87 and LN229 cell lines treated with PDIA4 siRNA, P4HB siRNA and negative control.

Supplementary Tables

Supplementary Table 1.

Characteristics of patients in cluster 1 and cluster 2 in CGGA dataset

Characteristic	N	Cluster 1	Cluster 2	p value
**Total cases**	310	132	178	
**Gender**				0.3377
Male	195	79	116	
Female	115	53	62	
**Age (years)**				0.0041
≤40	133	69	64	
＞40	177	63	114	
**Grade**				<0.0001
II	105	85	20	
III	67	29	38	
IV	138	18	120	
**Subtype**				<0.0001
Classical	70	13	57	
Mesenchymal	65	2	63	
Proneural	99	47	52	
Neural	76	70	6	
**IDH Status**				<0.0001
Mutation	160	100	60	
Wildtype	150	32	118	
**MGMT Promoter**				0.3125
Methylation	134	56	78	
Unmethylation	110	39	71	
**1p19q**				0.0001
Codel	36	26	10	
Non-codel	218	83	135	
**Chemotherapy**				0.0068
Yes	155	55	100	
No	124	64	60	
**Radiotherapy**				0.0898
Yes	207	98	109	
No	80	29	51	

Supplementary Table 2. ER stress related gene list							
ACADVL	BRSK2	DNAJB12	FCGR2B	MAP3K5	PTPN1	SSR1	TTC23L
ADD1	CALR	DNAJB14	FGF21	MBTPS1	PTPN2	STC2	TXNDC12
AGR2	CASP4	DNAJB2	FICD	MBTPS2	RASGRF1	STT3B	UBA5
AIFM1	CAV1	DNAJB9	FKBP14	NCK1	RASGRF2	STUB1	UBAC2
ALOX15	CCDC47	DNAJC10	FLOT1	NCK2	RCN3	SULT1A3	UBE2G2
AMFR	CCL2	DNAJC18	FOXRED2	NFE2L1	RHBDD1	SVIP	UBE2J1
ANKS4B	CCND1	DNAJC3	GET4	NFE2L2	RNF103	SYVN1	UBE2J2
ANKZF1	CDK5RAP3	EDEM1	GFPT1	NPLOC4	RNF121	TARDBP	UBE2K
APAF1	CEBPB	EDEM2	GORASP2	NRBF2	RNF139	TATDN2	UBE4A
ARFGAP1	CFTR	EDEM3	GOSR2	OPA1	RNF175	TBL2	UBE4B
ASNA1	CHAC1	EEF2	GRINA	OS9	RNF183	THBS1	UBQLN1
ASNS	CLU	EIF2AK2	GSK3A	P4HB	RNF185	THBS4	UBQLN2
ATF3	COL4A3BP	EIF2AK3	GSK3B	PARK7	RNF186	TLN1	UBXN1
ATF4	COPS5	EIF2AK4	HDGF	PARP16	RNF5	TMBIM6	UBXN4
ATF6B	CREB3L1	EIF2B5	HERPUD1	PDIA2	RNFT1	TMCO1	UBXN6
ATF6	CREB3L2	EIF2S1	HM13	PDIA3	SCAMP5	TMEM117	UBXN8
ATG10	CREB3L3	EP300	HSP90B1	PDIA4	SDF2L1	TMEM129	UFC1
ATP2A1	CREB3L4	ERLEC1	HSPA13	PDIA5	SDF2	TMEM33	UFM1
ATP2A2	CREB3	ERLIN1	HSPA1A	PDIA6	SEC16A	TMEM67	UGGT1
ATP6V0D1	CTDSP2	ERLIN2	HSPA5	PDX1	SEC31A	TMTC3	UGGT2
ATXN3	CTH	ERN1	HYOU1	PIK3R1	SEC61B	TMUB1	USP13
AUP1	CUL7	ERN2	IGFBP1	PIK3R2	SEL1L	TMUB2	USP14
BAK1	CXXC1	ERP27	ITPR1	PLA2G6	SERINC3	TMX1	USP19
BAX	DAB2IP	ERP29	JKAMP	PMAIP1	SERP1	TNFRSF10B	USP25
BBC3	DCTN1	ERP44	JUN	PML	SERP2	TOR1A	VAPB
BCAP31	DDIT3	EXTL1	KDELR3	POMT2	SESN2	TP53	VCP
BCL2L11	DDRGK1	EXTL2	KLHDC3	PPP1R15A	SGTA	TPP1	WFS1
BCL2L1	DDX11	EXTL3	LMNA	PPP1R15B	SGTB	TRAF2	WIPI1
BCL2	DERL1	FAF2	LRRK2	PPP2CB	SHC1	TRIB3	XBP1
BFAR	DERL2	FAM129A	MAGEA3	PPP2R5B	SIRT1	TRIM13	YIF1A
BHLHA15	DERL3	FBXO2	MAN1B1	PREB	SRPRB	TRIM25	YOD1
BOK	DNAJB11	FBXO6	MANF	PSMC6	SRPX	TSPYL2	ZBTB17

Supplementary Table 3.

Univariate Cox analysis of ER stress-related genes

Gene	HR	Low95	High95	P-value
P4HB	3.853964	3.194749	4.649204	4.06E-45
CASP4	2.060799	1.854444	2.290115	3.90E-41
EDEM2	3.165615	2.670171	3.752988	3.47E-40
PDIA4	2.865082	2.450395	3.349949	9.34E-40
PDIA6	3.572629	2.951084	4.325081	5.78E-39
TBL2	4.143845	3.346902	5.130551	6.76E-39
SHC1	2.218424	1.967887	2.500857	8.06E-39
DNAJB12	0.1122	0.080628	0.156135	1.70E-38
SIRT1	0.230743	0.184752	0.288182	3.05E-38
PLA2G6	0.377721	0.325799	0.437918	4.26E-38
KDELR3	1.572906	1.46704	1.686411	3.53E-37
WIPI1	2.834096	2.395594	3.352863	5.98E-34
CALR	3.859059	3.090329	4.819013	9.93E-33
FCGR2B	1.413263	1.334385	1.496803	3.69E-32
RNFT1	2.55893	2.175415	3.010057	8.15E-30
ERP44	2.73714	2.297285	3.261214	1.95E-29
PTPN2	3.342872	2.707826	4.126849	3.01E-29
BAK1	3.593299	2.871122	4.497126	5.51E-29
UBXN1	0.233887	0.180852	0.302474	1.68E-28
MANF	2.746997	2.296271	3.286193	2.17E-28
PDIA5	1.79805	1.619976	1.995699	2.86E-28
SERP2	0.472897	0.413885	0.540324	3.35E-28
RASGRF1	0.728796	0.688427	0.771532	1.42E-27
ADD1	0.175958	0.12864	0.240682	1.57E-27
HSPA5	3.159553	2.566442	3.889733	2.10E-27
TXNDC12	3.70597	2.922775	4.699033	2.94E-27
TOR1A	5.87437	4.257646	8.105	4.22E-27
CUL7	2.001318	1.763777	2.27085	5.17E-27
DERL2	4.035137	3.128968	5.203738	5.90E-27
FAM129A	1.66412	1.515546	1.82726	1.35E-26
BCAP31	5.052964	3.746269	6.815434	2.64E-26
SCAMP5	0.542857	0.483805	0.609117	2.56E-25
DAB2IP	0.378686	0.315259	0.454875	2.99E-25
BRSK2	0.66714	0.618027	0.720156	3.24E-25
PIK3R1	0.486135	0.423409	0.558153	1.41E-24
HM13	3.376275	2.673773	4.263351	1.57E-24
SRPRB	2.956089	2.399947	3.641106	2.14E-24
XBP1	2.744248	2.254946	3.339724	7.10E-24
EEF2	0.304555	0.241377	0.384269	1.21E-23
PREB	4.356979	3.264506	5.81505	1.64E-23
UBQLN2	0.193438	0.139995	0.267283	2.33E-23

LMNA	2.283677	1.940998	2.686854	2.40E-23
SEC61B	3.268575	2.572476	4.153035	3.23E-22
RCN3	1.656728	1.495081	1.835852	5.52E-22
THBS1	1.328443	1.253789	1.407542	6.31E-22
DERL3	1.733509	1.548299	1.940875	1.39E-21
ERP27	1.480476	1.365231	1.60545	2.33E-21
GOSR2	6.328056	4.321794	9.265664	2.47E-21
BAX	2.564453	2.101995	3.128655	1.66E-20
UBE2J2	3.41657	2.633988	4.431663	2.10E-20
PPP1R15A	1.961259	1.699778	2.262965	2.80E-20
TSPYL2	0.51856	0.450959	0.596295	3.12E-20
HSP90B1	3.388866	2.606211	4.406557	8.28E-20
PTPN1	5.166393	3.62755	7.358029	8.87E-20
BFAR	4.398289	3.186995	6.069965	2.02E-19
DCTN1	0.366356	0.294399	0.455901	2.25E-19
TMUB1	2.292443	1.91175	2.748945	3.43E-19
TMEM67	1.823825	1.59736	2.082397	6.48E-19
CAV1	1.456275	1.338712	1.584162	2.09E-18
MAP3K5	0.687224	0.631827	0.747477	2.18E-18
DNAJB11	3.188682	2.452981	4.145036	4.53E-18
OPA1	0.289204	0.218268	0.383193	5.58E-18
ATF3	1.507305	1.372127	1.6558	1.14E-17
KLHDC3	0.251728	0.183427	0.345462	1.33E-17
TMCO1	3.933461	2.872253	5.386754	1.38E-17
CEBPB	1.796624	1.567576	2.059139	3.75E-17
ATG10	2.603721	2.077861	3.262666	9.28E-17
AUP1	2.844334	2.221711	3.641442	1.10E-16
EXTL3	2.69778	2.127512	3.420906	2.59E-16
EP300	0.523533	0.448238	0.611476	3.12E-16
TNFRSF10B	1.957619	1.665039	2.301611	4.19E-16
DNAJC10	3.329586	2.487735	4.456319	6.05E-16
SDF2L1	2.038647	1.715083	2.423254	6.59E-16
FLOT1	2.758143	2.155182	3.529796	7.57E-16
EXTL2	2.732572	2.139761	3.489618	7.84E-16
ASNA1	2.255765	1.846983	2.755022	1.53E-15
TMBIM6	4.068423	2.878033	5.751175	1.93E-15
ERP29	3.098944	2.341647	4.101155	2.54E-15
RNF103	0.179079	0.116854	0.274439	2.87E-15
TRIM13	0.339027	0.259186	0.443463	2.91E-15
CCL2	1.313213	1.22675	1.405769	4.46E-15
EDEM1	2.959507	2.24564	3.900306	1.32E-14
THBS4	1.410405	1.292037	1.539617	1.48E-14
TMTC3	1.851332	1.578093	2.171881	4.05E-14
PML	2.587797	2.017702	3.318969	6.96E-14

OS9	1.540746	1.375613	1.725702	7.82E-14
DNAJB9	2.153395	1.759304	2.635765	1.02E-13
PDIA3	2.61744	2.030679	3.373745	1.09E-13
FBXO6	1.789962	1.529764	2.094416	3.75E-13
DDIT3	1.51712	1.35508	1.698537	4.74E-13
PDIA2	0.765206	0.71111	0.823417	8.43E-13
DNAJC18	0.396144	0.306256	0.512414	1.76E-12
CTDSP2	1.695182	1.462664	1.964662	2.35E-12
NPLOC4	4.855602	3.116513	7.565143	2.86E-12
UBE2G2	0.304916	0.217606	0.427258	5.18E-12
JUN	1.651171	1.430994	1.905226	6.52E-12
TMX1	2.542912	1.945029	3.324579	8.81E-12
UBXN8	2.870791	2.119045	3.889223	9.92E-12
UBE4A	0.26575	0.180599	0.39105	1.77E-11
EIF2AK4	3.023458	2.189957	4.17419	1.77E-11
FBXO2	0.765275	0.707113	0.828222	3.28E-11
DERL1	2.862918	2.091724	3.918444	5.08E-11
NCK1	2.80892	2.058028	3.833783	7.63E-11
SDF2	2.403138	1.838772	3.140722	1.36E-10
RNF185	0.276538	0.185594	0.412046	2.66E-10
DDRGK1	2.529138	1.893826	3.377573	3.24E-10
CHAC1	1.400449	1.260352	1.556119	3.78E-10
DNAJB2	0.467401	0.367683	0.594163	5.23E-10
GSK3B	0.484761	0.385435	0.609683	6.03E-10
SEC31A	2.409759	1.816973	3.195942	1.03E-09
NFE2L2	3.278393	2.238066	4.802299	1.09E-09
USP13	0.391509	0.28953	0.529407	1.12E-09
RASGRF2	0.722972	0.651167	0.802694	1.22E-09
ATXN3	0.375147	0.273351	0.514853	1.28E-09
TRIB3	1.427584	1.272357	1.60175	1.35E-09
USP14	2.699096	1.940602	3.754052	3.66E-09
GRINA	0.447578	0.341929	0.585871	4.86E-09
FOXRED2	0.476445	0.371597	0.610876	5.02E-09
PSMC6	0.333847	0.230969	0.48255	5.33E-09
CREB3L2	1.885026	1.523076	2.332992	5.62E-09
FAF2	3.637522	2.345016	5.64242	8.16E-09
TMUB2	3.875167	2.440796	6.152467	9.28E-09
COPS5	2.711918	1.929283	3.812038	9.33E-09
RNF5	0.48563	0.378921	0.62239	1.16E-08
EXTL1	0.733367	0.659178	0.815905	1.21E-08
TP53	1.759926	1.445538	2.14269	1.80E-08
SEC16A	0.456184	0.342563	0.60749	7.86E-08
GET4	2.341863	1.710172	3.206883	1.12E-07
BCL2	0.63412	0.53525	0.751254	1.39E-07

ASNS	1.702518	1.391216	2.083477	2.41E-07
SSR1	3.410561	2.137195	5.442614	2.68E-07
TTC23L	1.349094	1.201107	1.515313	4.39E-07
EIF2AK3	2.018027	1.530626	2.660632	6.43E-07
UBAC2	2.440327	1.711063	3.480406	8.43E-07
UBA5	0.328506	0.209722	0.514567	1.16E-06
ERN2	1.543305	1.29176	1.843833	1.75E-06
GFPT1	0.451475	0.32504	0.627092	2.10E-06
USP19	0.477803	0.351064	0.650297	2.65E-06
SESN2	1.649293	1.332589	2.041265	4.24E-06
CREB3L1	1.168076	1.092477	1.248906	5.34E-06
COL4A3BP	0.487844	0.356013	0.668492	7.99E-06
HYOU1	2.104219	1.517847	2.917116	8.05E-06
DNAJC3	2.164287	1.541289	3.039105	8.28E-06
NCK2	1.905561	1.433717	2.53269	8.92E-06
SRPX	1.259817	1.136122	1.396979	1.19E-05
CLU	1.382935	1.195918	1.599199	1.22E-05
SGTB	0.596438	0.473063	0.75199	1.24E-05
SEL1L	0.575932	0.44938	0.738123	1.31E-05
CREB3L3	0.705281	0.602077	0.826175	1.52E-05
PPP2R5B	0.494055	0.358807	0.680284	1.56E-05
PARP16	1.905095	1.421195	2.553759	1.63E-05
USP25	0.525337	0.391698	0.704572	1.72E-05
BCL2L1	1.938785	1.431133	2.626511	1.92E-05
PARK7	1.858102	1.394487	2.475852	2.33E-05
MAN1B1	1.88519	1.397978	2.542201	3.24E-05
GORASP2	2.503962	1.596643	3.92688	6.39E-05
MAGEA3	1.279469	1.132524	1.445481	7.52E-05
TPP1	1.62771	1.27705	2.074658	8.30E-05
ARFGAP1	0.558138	0.417208	0.746672	8.59E-05
CFTR	0.771461	0.677497	0.878458	9.02E-05
CREB3L4	0.706556	0.593283	0.841455	9.77E-05
SYVN1	0.436995	0.288126	0.662784	9.81E-05
EIF2AK2	1.478893	1.21015	1.807317	0.000131
TRIM25	1.664628	1.276971	2.169968	0.000165
PIK3R2	0.596827	0.455435	0.782115	0.000183
ACADVL	1.643808	1.266867	2.132904	0.000184
GSK3A	1.804888	1.323336	2.461672	0.000192
ATP2A2	0.597113	0.455359	0.782996	0.000192
YOD1	0.583394	0.435331	0.781816	0.000309
EDEM3	0.580764	0.431631	0.781424	0.000332
FKBP14	1.463955	1.184584	1.809213	0.000419
POMT2	0.583773	0.432373	0.788186	0.000442
UBXN4	2.20004	1.404284	3.446723	0.000577

FGF21	1.665145	1.238357	2.239021	0.000738
UBE4B	0.62584	0.474119	0.826112	0.000938
ERLEC1	1.91821	1.303801	2.822157	0.000944
CREB3	1.752651	1.248684	2.460019	0.001179
LRRK2	1.207116	1.076036	1.354163	0.00133
ATP6V0D1	0.560554	0.385542	0.815009	0.002436
ANKZF1	0.706171	0.563036	0.885695	0.00261
NRBF2	1.643121	1.181895	2.284337	0.003136
DNAJB14	0.734964	0.598974	0.901828	0.00318
ERLIN1	1.743986	1.191619	2.552397	0.004208
CCND1	0.838782	0.742188	0.947947	0.004858
HERPUD1	1.468653	1.123392	1.920027	0.00494
STUB1	0.607098	0.428005	0.861131	0.005138
WFS1	0.756124	0.616413	0.927501	0.007318
ATF6B	0.563322	0.36924	0.85942	0.007747
HDGF	1.624704	1.13487	2.325962	0.008024
STT3B	1.407856	1.089156	1.81981	0.008998
RHBDD1	1.546828	1.088164	2.198818	0.015065
UFC1	1.585949	1.086423	2.315151	0.016875
UGGT1	1.557133	1.078106	2.249004	0.018231
TRAF2	1.464986	1.059122	2.026381	0.021055
RNF175	1.111433	1.015574	1.21634	0.021689
AGR2	0.819997	0.688128	0.977137	0.026519
HSPA1A	0.873609	0.774446	0.98547	0.027944
FICD	0.815336	0.677163	0.981703	0.031171
CCDC47	1.626975	1.044495	2.534285	0.031359
JKAMP	1.516035	1.023835	2.244855	0.037748
EIF2S1	1.461937	1.018125	2.099211	0.03966
SVIP	0.865971	0.754398	0.994045	0.04087
RNF183	0.853403	0.730704	0.996704	0.045322
MBTPS1	0.636564	0.408815	0.991192	0.045595
PDX1	1.083398	1.001221	1.172319	0.046558
VCP	1.703621	1.001717	2.897353	0.049265
TMEM117	1.298863	1.000783	1.685726	0.049316
STC2	0.917428	0.841685	0.999986	0.049964
DDX11	1.131991	0.986484	1.298961	0.077377
ZBTB17	0.771653	0.57647	1.032923	0.081465
SULT1A3	1.148357	0.979851	1.345841	0.087533
BOK	0.929795	0.854197	1.012084	0.092501
ANKS4B	0.766861	0.559685	1.050726	0.098531
MBTPS2	0.788591	0.592041	1.050393	0.104413
UBXN6	0.734947	0.50668	1.066053	0.104613
AIFM1	1.417109	0.929039	2.161586	0.1056
EIF2B5	0.651393	0.382148	1.110338	0.115185

SGTA	0.742474	0.507408	1.086439	0.125248
IGFBP1	1.065181	0.982074	1.155321	0.127627
PMAIP1	1.097863	0.968958	1.243917	0.142886
TLN1	1.195408	0.932622	1.532238	0.158769
CTH	1.133039	0.951969	1.348551	0.159751
PPP1R15B	1.151972	0.94025	1.411369	0.172136
CDK5RAP3	0.854497	0.679191	1.075051	0.179523
TMEM33	1.407397	0.852909	2.322365	0.18111
ERLIN2	1.242966	0.892951	1.730179	0.197409
HSPA13	1.188664	0.902407	1.565724	0.218899
BBC3	1.109722	0.921389	1.33655	0.272574
RNF121	0.773012	0.48373	1.235292	0.281718
UFM1	1.246169	0.8186	1.897064	0.304693
NFE2L1	0.863325	0.649273	1.147944	0.312061
VAPB	0.809641	0.52964	1.237668	0.329454
APAF1	0.868018	0.628927	1.198001	0.389225
ATF4	1.135571	0.839582	1.535909	0.409291
TATDN2	1.255444	0.729312	2.161133	0.411698
BCL2L11	0.931354	0.780219	1.111765	0.431169
CXXC1	0.875377	0.610998	1.254153	0.468128
ITPR1	1.024105	0.926117	1.132461	0.642522
ATP2A1	1.024952	0.915527	1.147456	0.668756
SERP1	0.92189	0.623279	1.363563	0.683837
PPP2CB	0.940308	0.690631	1.280247	0.695871
TMEM129	1.062487	0.761105	1.483211	0.721756
ERN1	0.978529	0.868088	1.103021	0.722425
ALOX15	1.016185	0.920923	1.121301	0.749208
YIF1A	1.045555	0.787539	1.388102	0.758009
BHLHA15	1.020259	0.887827	1.172446	0.77738
RNF186	1.075377	0.598608	1.931877	0.807901
UBE2J1	0.954763	0.633818	1.438223	0.824737
ATF6	1.033881	0.757187	1.411685	0.833925
UBE2K	1.043205	0.701292	1.551815	0.834641
UBQLN1	0.953804	0.604542	1.504845	0.838904
AMFR	0.972452	0.666109	1.419684	0.884948
TARDBP	0.959842	0.540963	1.703067	0.888585
SERINC3	0.988838	0.738139	1.324684	0.940023
UGGT2	1.002954	0.768135	1.309558	0.982707
RNF139	1.000433	0.708254	1.413146	0.998039

Supplementary Table 4.

Univariate and multivariate analysis of OS in CGGA sequencing dataset

Variables	Univariate analysis		Multivariate analysis	
	HR (95% CI)	p value	HR (95% CI)	p value
**Risk score**	7.227 (5.074-10.229)	< 0.001	2.300 (1.072-4.932)	0.032
**Age at Diagnosis**	1.038 (1.023-1.054)	< 0.001	1.001 (0.980-1.023)	0.938
**Gender**	1.181 (0.837-1.666)	0.345	-	-
**WHO Grade**	3.477 (2.716-4.452)	< 0.001	2.072 (1.404-3.058)	< 0.001
**TCGA Subtype**	1.935 (1.641-2.282)	< 0.001	1.160 (0.867-1.551)	0.318
**IDH mutation status**	0.228 (0.158-0.329)	< 0.001	0.952 (0.448-2.026)	0.899
**MGMT methylation**	0.528 (0.374-0.450)	< 0.001	0.791 (0.503-1.242)	0.309
**1p/19q co-deletion**	0.134 (0.049-0.363)	< 0.001	0.544 (0.189-1.567)	0.260
**Radiotherapy**	0.429 (0.296-0.622)	< 0.001	0.402 (0.259-0.623)	< 0.001
**Chemotherapy**	1.378 (0.963-1.971)	0.079	-	-
